# Bioinspired Simultaneous Learning and Motion–Force Hybrid Control for Robotic Manipulators Under Multiple Constraints

**DOI:** 10.3390/biomimetics10120841

**Published:** 2025-12-15

**Authors:** Yuchuang Tong, Haotian Liu, Zhengtao Zhang

**Affiliations:** 1 CAS Engineering Laboratory for Intelligent Industrial Vision, Institute of Automation, Chinese Academy of Sciences, Beijing 100190, China; yuchuang.tong@ia.ac.cn (Y.T.); liuhaotian2021@ia.ac.cn (H.L.); 2 Beijing Zhongke Huiling Robot Technology Co., Ltd., Beijing 100192, China

**Keywords:** bioinspired control, robotic manipulators, motion–force coordination, learning-based hybrid control, multiple constraint limitations

## Abstract

Inspired by the adaptive flexible motion coordination of biological systems, this study presents a bioinspired control strategy that enables robotic manipulators to achieve precise and compliant motion–force coordination for embodied intelligence and dexterous interaction in physically constrained environments. To this end, a learning-based motion–force hybrid control (LMFC) framework is proposed, which unifies learning and kinematic-level control to regulate both motion and interaction forces under incomplete or implicit kinematic information, thereby enhancing robustness and precision. The LMFC formulation recasts motion–force coordination as a time-varying quadratic programming (TVQP) problem, seamlessly incorporating multiple practical constraints—including joint limits, end-effector orientation maintenance, and obstacle avoidance—at the acceleration level, while determining control decisions at the velocity level. An RNN-based controller is further designed to integrate adaptive learning and control, enabling online estimation of uncertain kinematic parameters and mitigating joint drift. Simulation and experimental results demonstrate the effectiveness and practicality of the proposed framework, highlighting its potential for adaptive and compliant robotic control in constraint-rich environments.

## 1. Introduction

Biological organisms exhibit dexterous, compliant, and resilient manipulation and locomotion through the tightly coupled integration of perception, actuation, and adaptive learning, enabling continuous regulation of both motion and interaction forces under dynamically changing environmental constraints. Inspired by these principles, roboticists seek to imbue manipulators with analogous embodied intelligence, whereby motion–force coordination is achieved in a compliant, adaptive, and robust manner [1,2,3].

Robotic manipulators are increasingly deployed across industrial, aerospace, medical, and defense applications, frequently operating in scenarios that demand continuous interaction within complex and uncertain environments. In such tasks, precise trajectory tracking alone is insufficient; adaptive regulation of interaction forces and constraint-aware motion planning are equally critical to ensure safety, stability, and task effectiveness. The central challenge, therefore, lies in replicating the flexible, adaptive, and robust coordination mechanisms characteristic of biological systems, which seamlessly integrate sensing, actuation, and learning to enable real-time adjustment to dynamic contexts [4,5,6]. In this regard, admittance control frameworks have been widely explored as early attempts to emulate biological compliance, enhancing environmental adaptability and robustness against modeling uncertainties [7,8,9].

Achieving bioinspired dexterity and adaptability in robotic manipulation requires control strategies that integrate precision, flexibility, and multi-level constraint management, including obstacle avoidance, end-effector orientation maintenance, and adherence to physical limitations [10,11]. Conventional controllers often emphasize positional accuracy at the expense of orientation or posture fidelity [12]. Optimization-based methods can improve orientation control but typically rely on highly accurate models, limiting generalization and real-time performance [13,14]. Physical constraints may also introduce excessive joint accelerations or discontinuities, potentially damaging mechanical components [15,16,17,18,19]. Real-time enforcement of such constraints remains computationally demanding [20,21,22], motivating bioinspired methods that integrate acceleration-level and velocity-level considerations to achieve embodied, compliant, and robust motion control.

In practical operations, kinematic inaccuracies frequently arise from prolonged use, structural wear, or manufacturing variances [23]. Biological systems overcome such uncertainties through adaptive internal model refinement, continuously updating motor commands based on sensory feedback. Translating this principle to robotics, data-driven and learning-based control strategies can compensate for kinematic uncertainties in real time, enhancing robustness, accuracy, and overall manipulation performance [24,25,26,27]. Effective obstacle avoidance is another essential aspect of embodied robotic behavior, reflecting how biological agents continuously integrate spatial perception and motion planning when navigating cluttered or dynamic environments [28,29]. Traditional methods often rely primarily on geometric distance measures, neglecting dynamic task constraints [12,30,31]. This highlights the need for holistic bioinspired frameworks capable of simultaneously handling multiple practical constraints within a unified control structure. In repetitive motion planning (RMP) tasks, cumulative joint drift poses a persistent challenge, potentially degrading task accuracy and inducing mechanical stress [21,32]. Existing joint-drift-free (JDF) schemes struggle to reconcile joint-level stability with task-space performance due to inherent coupling [33]. A bioinspired solution should therefore aim to decouple joint drift from end-effector deviations, while preserving compliant and adaptive task execution, analogous to the way biological motor systems distribute and compensate for internal and external perturbations.

**Figure 1:**
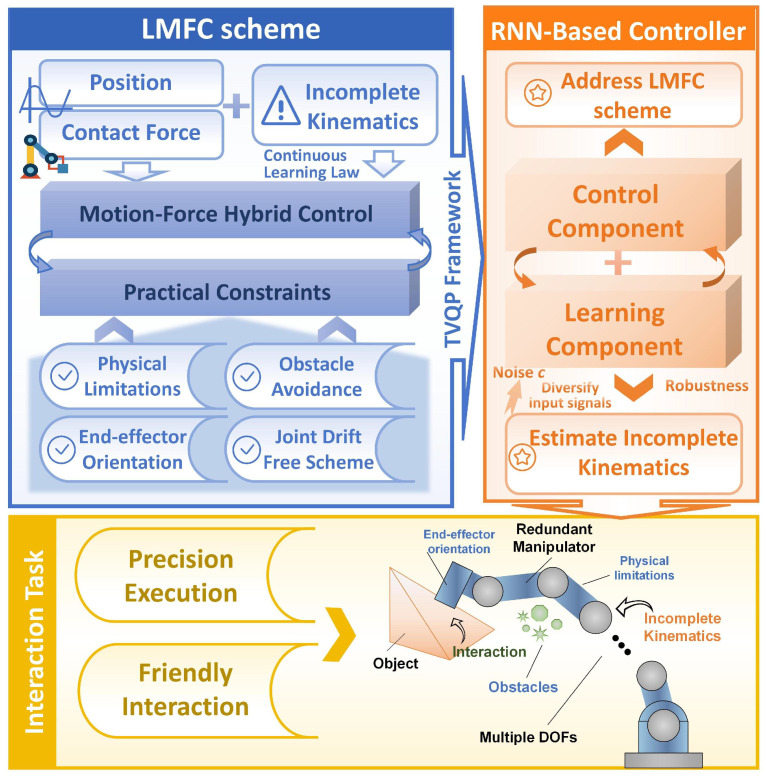
The organisational framework of this paper.

Motivated by these bioinspired principles, this paper proposes a simultaneous learning and motion–force hybrid control (LMFC) framework for robotic manipulators, addressing challenges such as incomplete kinematic information and multiple practical constraints, including physical limits, end-effector orientation maintenance, obstacle avoidance, and joint drift correction. Figure 1 illustrates the system architecture, where the term “bioinspired” refers to the conceptual principle that, analogous to biological systems, the framework tightly integrates perception, learning, and actuation to achieve adaptive, compliant, and robust motion–force coordination under dynamically changing constraints. Table 1 demonstrates LMFC’s superior capability in satisfying multi-level constraints, achieving precise motion–force coordination, and handling uncertain kinematics, surpassing existing state-of-the-art (SOTA) methods [4,7,10,12,13,14,15,16,17,18,20,21,32,33,34,35]. The technical novelty of this paper lies in the systematic integration of individually established components into a unified framework that simultaneously handles incomplete kinematic information while enforcing multi-level practical constraints, rather than merely combining known techniques.

Specifically, unlike conventional methods [4,7,10,12,14,16,17,18,20,21,32,33,35] that primarily focus on motion control while neglecting uncertain kinematics or multi-objective optimization, LMFC integrates both motion and force regulation, dynamically adapting to incomplete kinematic information while simultaneously enforcing constraints on obstacles, physical limits, orientation, and joint drift. In contrast to other hybrid motion–force methods [13,15,34], LMFC employs an RNN-based controller for online estimation of implicit kinematic parameters, enhancing robustness and applicability in dynamic, unstructured environments. Its kinematic-level formulation further distinguishes LMFC from conventional dynamics-based strategies, such as impedance or admittance control [36]. By combining acceleration-level constraints with velocity-level decision variables, the framework effectively addresses the unconventional time-varying quadratic programming (TVQP) problem, while decoupling joint drift from end-effector deviations to improve precision, stability, and compliance, outperforming existing repetitive motion planning schemes [21,32,33]. Moreover, the RNN-based design provides superior temporal consistency and more reliable online adaptation to uncertain, time-varying kinematics, resulting in significantly enhanced robustness in dynamic environments [37,38]. Overall, the proposed LMFC framework embodies a bioinspired method that tightly integrates learning and control to achieve robust, adaptive, and constraint-aware motion–force coordination, ensuring precise, compliant, and safe manipulation in complex, constraint-rich scenarios. The main contributions of this work are summarized below.

1. We introduce a bioinspired LMFC framework that emulates adaptive coordination in biological systems, integrating kinematic-level control with online learning to achieve precise, robust, and compliant motion–force regulation under incomplete kinematic knowledge and multiple practical constraints.
2. Drawing inspiration from decoupled biological actuation, LMFC explicitly separates joint drift from end-effector deviations and combines acceleration-level constraints with velocity-level decision variables, enabling stable, constraint-aware motion–force execution with maintained orientation and effective obstacle avoidance.
3. We design an RNN-based adaptive controller, motivated by sensorimotor learning, to perform online estimation of unknown or implicit kinematic parameters, providing dynamic adaptation and reliable enforcement of multi-level constraints in unstructured and uncertain environments.

The remainder of this paper is organized as follows. Section 2 reviews the preliminary concepts and problem formulation. Section 3 presents the proposed LMFC scheme within a TVQP framework. Section 4 introduces an RNN-based adaptive controller for real-time implementation of the scheme. Section 5 validates the proposed method through comprehensive simulations and physical experiments. Section 6 concludes the paper and discusses potential directions for future work.

## 2. Preliminaries and Problem Formulation

The preliminaries and problem formulation are introduced, primarily addressing learning laws for unknown kinematics and multi-constraint limitations, which form the foundation for the scheme formulation and controller design.

### 2.1. Kinematics of Robotic Manipulators

This section outlines the kinematic modeling of robotic manipulators and the trajectory tracking task. First, the forward kinematics equation of robotic manipulators is

(1) $$\begin{array}{c} r=f(q) \end{array}$$

where $q\left(t\right)\in R^{n}$ is the joint angle vector, $r\left(t\right)\in R^{m}\left(m\leq n\right)$ is end-effector position, *m* and *n* denote the dimensions of the task space and joint space, respectively. For the convenience of description, the parameter *t* is omitted in the following part.

Generally, to solve the nonlinear function $f\left(\;\cdot \;\right)\in R^{m}$, it is converted into a linear equation in velocity level, i.e.,

(2) $$\begin{array}{c} J\left(q\right)\dot{q}=\dot{r} \end{array}$$

where $\dot{r}\in R^{m}$ and $\dot{q}\in R^{n}$ represent vector of the end-effector velocity and joint angle velocity, respectively, $J=\partial f/\partial q\in R^{m\times n}$ is defined as Jacobian matrix.

The trajectory tracking task for robotic manipulators focuses on achieving a high level of accuracy by ensuring that the actual position and velocity of the end-effector closely approximate the desired values, i.e., $r_{a}\to r_{d}$ and ${\dot{r}}_{a}\to {\dot{r}}_{d}$, which is further designed as

(3) $$\begin{array}{c} J\dot{q}+\gamma \left(r_{d}-r_{a}\right)={\dot{r}}_{d} \end{array}$$

where the coefficient γ>0 scales the convergence rate. The actual position and velocity of the end-effector, i.e., $r_{a}$ and ${\dot{r}}_{a}$, can be determined by utilizing feedback from position sensors installed at the end-effector or by acquiring measurements from external devices, such as laser trackers.

### 2.2. Learning Law for Unknown Kinematics

Accurate kinematic modeling is essential for achieving precise robot control. However, uncertainties in kinematic parameters, caused by prolonged use or deviations from factory specifications, often pose challenges. To address this, this section explores learning-based methods for estimating unknown kinematics. The estimated Jacobian matrix J^ is introduced, reformulating the kinematic equations as

(4) J^q˙=r˙^a

where r˙^a denotes the estimated end-effector velocity, calculated based on J^.

According to the gradient descent method, we get

(5) J^i+1=J^i−ϵ∇ψJ^i

where ψ=r˙^a−r˙a22/2 is defined as the error function, ϵ is the step size, *i* is the iteration index.

Then, the adaptation method is obtained as

(6) J^˙=J^i+1−J^iρ=−ϵ∇ψJ^iρ=−ϵρr˙^ai−r˙aiq˙T

where ρ>0, J^˙ is the derivative of J^.

Thus, the learning method is further reorganised as

(7) ιJ^˙=r˙a−J^q˙q˙T

where ι=ρ/ϵ≪1 is the iteration step size.

**Remark** **1.** *As ι decreases, the estimation accuracy of J^ improves. Real-time measurements of end-effector and joint velocities are used for data-driven learning, resulting in a convex problem for the Jacobian matrix estimation. This convexity ensures coherence between local and global extrema, eliminating concerns about local extrema. This property instills confidence in achieving reliable control over manipulator position and orientation. Avoiding local extrema issues enhances robustness and consistent performance in manipulating the robot, contributing to the overall effectiveness and reliability in practical applications.*

### 2.3. Physical Constraints

Physical constraints are crucial for ensuring the safety, precision, and stability of robotic manipulators by preventing limit violations, optimizing energy use, and maintaining dynamic feasibility, this section defines these constraints as

(8a) $$q^{-}\leq q\leq q^{+}$$

(8b) $${\dot{q}}^{-}\leq \dot{q}\leq {\dot{q}}^{+}$$

(8c) $${\ddot{q}}^{-}\leq \ddot{q}\leq {\ddot{q}}^{+}$$

where $\ddot{q}$ denotes the joint angular acceleration, The symbol ^+^ and ^−^ denote the upper bounds and lower bounds, respectively.

To consolidate the various levels of physical constraints (8) into expressions associated with control variables, (8a) and (8b) are uniformly transformed into the velocity-level constraint, denoted as:

(9) $$\begin{array}{c} \vartheta^{-}\left(t\right)\leq \dot{q}\left(t\right)\leq \vartheta^{+}\left(t\right) \end{array}$$

where $\vartheta^{-}=\max\left\{{\dot{q}}^{-},\xi \left(q^{-}-q\right)\right\}$ and $\vartheta^{+}=\min\left\{{\dot{q}}^{+},\xi \left(q^{+}-q\right)\right\}$ with ξ>0.

Then, (9) can be further equivalently expressed as follows:

(10) $$\begin{array}{c} \dot{q}\left(t\right)\in \Theta \end{array}$$

where $\Theta =\{\dot{q}\in R^{n}|\vartheta^{-}\left(t\right)\leq \dot{q}\leq \vartheta^{+}\left(t\right)\}$.

It is guaranteed to be within range using the infinite-norm of the acceleration level constraint (8c), which is denoted as

(11) $$\begin{array}{c} ∥\ddot{q}{∥}_{\infty }\leq \eta_{\max} \end{array}$$

where $\eta_{\max}$ is defined as the minimum absolute value in ${\ddot{q}}^{-}$ and ${\ddot{q}}^{+}$.

According to the derivation of abovementioned Equations (8)–(11), the physical constraint of the robotic manipulator is finally expressed in the following form

(12) $$\begin{array}{cc} \dot{q}\left(t\right) & \in \Theta \\ ∥\ddot{q}{∥}_{\infty } & \leq \eta_{\max}. \end{array}$$

Hereto, the physical constraints at different levels in (8) are uniformly transformed into inequality constraints at velocity level and acceleration level, respectively.

### 2.4. Obstacle Avoidance Constraint

The obstacle avoidance constraint ensures safe and effective operation by preventing collisions and enabling the robot to function in dynamic, unstructured environments. In this section, obstacle avoidance is framed as preventing overlap between critical points and obstacle points, defined as a set of constraints. First, a vector ${\vec{p}}_{\mathrm{AB}}$ is defined as

(13) $$\begin{array}{c} {\vec{p}}_{\mathrm{AB}}=\mathrm{sgn}{\left[x_{A}-x_{B},y_{A}-y_{B},z_{A}-z_{B}\right]}^{T} \end{array}$$

where obstacle point B is defined as several simplified points where the obstacle with arbitrary shape is closest to the manipulator, critical point A is defined as the closest point on the link or its extension within the danger zone, the symbol sgn(·) is defined as a scalar signum function, and $\left(x_{A},y_{A},z_{A}\right)$ and $\left(x_{B},y_{B},z_{B}\right)$ are the coordinates of A and B, respectively.

Thus, the obstacle avoidance constraint is designed to be expressed in the form of a mathematical inequality as

(14) $$\begin{array}{c} J_{B}\dot{q}⩽0 \end{array}$$

with $J_{B}\in R^{ma\times n}$ denoting as

(15) $$\begin{array}{c} J_{B}=-{\vec{p}}_{\mathrm{AB}}⋄J_{A} \end{array}$$

where $J_{A}\in R^{ma\times n}$ represents the Jacobian matrix of A, *a* denotes pairs of critical points and effective obstacle points located on links within the danger zone. The danger zone refers to the region around the robotic manipulator where collisions with obstacles may occur. Its size can be adjusted for maneuvering precision or safety considerations. The operator ⋄ is denoted as $A⋄B={\left[A_{1}B_{1},A_{2}B_{2},\ldots ,A_{\ell }B_{\ell }\right]}^{T}$ with $A=\left[A_{1},A_{2},\ldots ,A_{\ell }\right]$ defining as a column vector and $B={\left[B_{1},B_{2},\ldots ,B_{\ell }\right]}^{T}$ defining as a matrix.

To mitigate abrupt motion changes near obstacles due to the null vector 0 in inequality (14), this paper suggests using a gradually changing variable μ for smoother motion and improved obstacle avoidance effectiveness. The adjusted expression is as follows:

(16) $$\begin{array}{c} J_{B}\dot{q}⩽\mu \end{array}$$

with variable $\mu \in R^{ma}$ defining as

(17) $$\begin{array}{c} \mu =F\left(s\right)\max\left\{{\left.J_{B}\dot{q}\right|}_{s=s_{a}},0\right\} \end{array}$$

and the smooth function F defining as

(18) $$\begin{array}{c} F\left(s\right)=\left\{\begin{array}{cc} 1 & \;\mathrm{if}\;s⩾s_{a}\\ \sin^{2}\left(\frac{\pi }{2}\;\cdot \;\frac{s-s_{o}}{s_{a}-s_{o}}\right) & \;\mathrm{if}\;s_{o}<s<s_{a}\\ 0 & \;\mathrm{if}\;s⩽s_{o} \end{array}\right. \end{array}$$

where $s_{o}$ is the safety distance based on link thickness to avoid collisions, $s_{a}$ is the activation distance for obstacle avoidance, and *s* is the minimum separation between point sets A and B for obstacle evaluation and avoidance determination.

**Remark** **2.** *The obstacle avoidance constraint (16) introduces an additional deviation velocity to maintain a safe distance and guide the links within the danger zone, ensuring obstacle avoidance. When the distance s is less than the activation threshold $s_{a}$, the obstacle avoidance function is activated, steering the manipulator away from the obstacle. Safety is assured with s above the safety threshold $s_{o}$, but lower values require a greater deviation velocity to avoid collision. For s between $s_{o}$ and $s_{a}$, a smooth deviation velocity is applied to enable the manipulator to move away from obstacles smoothly. Choosing the parameter $s_{a}$ balances safety and flexibility, with a higher value enhancing safety but potentially constraining flexibility and increasing computational requirements. Selecting an appropriate $s_{a}$, considering security and hardware capabilities, is essential for achieving the optimal trade-off between safety and flexibility in practical scenarios.*

### 2.5. End-Effector Orientation Maintenance Constraint

In practical applications, maintaining consistent and precise end-effector orientation is essential, and incorporating arbitrary installations in the control scheme enhances versatility. This section treats end-effector orientation preservation as a constraint governing the workspace of arbitrarily mounted end-effectors, expressed as

(19) $$\begin{array}{c} R(t)=R(0) \end{array}$$

where the rotation matrix R=[n,o,a] represents the orientation of the end-effector in relation to the base coordinate system, in which $n={\left[n_{1},n_{2},n_{3}\right]}^{T}$, $o={\left[o_{1},o_{2},o_{3}\right]}^{T}$ and $a={\left[a_{1},a_{2},a_{3}\right]}^{T}$ denoting the column vectors comprising the matrix R. R(0)∈SO(3) corresponds to the initial state of the end-effector’s orientation.

The orthogonality of the rotation matrix R allows for determining row or column vectors by fixing any two elements, leading to the following direction form as

(20) $$\begin{array}{c} \varpi ={\left[o_{2},o_{3},a_{2},a_{3}\right]}^{T} \end{array}$$

In turn, ϖ is converted into the velocity layer, for which the derivative gives $\dot{\varpi }\left(0\right)=\dot{\varpi }\left(t\right)$. Thus it follows that

(21) $$\begin{array}{c} M\dot{q}=0 \end{array}$$

where M=∂ϖ/∂q is the Jacobian matrix of ϖ. The paper illustrates mounting the end-effector vertically on a manipulator relative to the task plane, with this mounting considered arbitrary due to the manipulator’s flexibility.

## 3. Scheme Formulation

Motivated by bioinspired principles of integrated sensorimotor coordination, this section unifies physical limitations, obstacle avoidance, and end-effector orientation maintenance constraints within the motion–force control and enhanced JDF criteria, reformulating them as a cohesive multi-objective TVQP framework to implement the LMFC scheme.

### 3.1. Motion–Force Control Criterion

This section examines the motion–force control criterion from a kinematic perspective, focusing on the challenge of controlling motion and contact forces during the manipulator’s interaction with an object, as depicted in Figure 1.

During contact, we introduce the diagonal matrix $\Gamma =\mathrm{diag}(\Gamma_{x},\Gamma_{y},\Gamma_{z})$, which represents the decoupling of contact forces and tracking errors along the different axes of the end-effector’s coordinate system. Each element of the matrix independently influences its respective axis. Consequently, the relationship between the contact force and displacement is given by the following expression:

(22) $$\begin{array}{c} f_{e}=\varepsilon \Gamma d_{e} \end{array}$$

Here, $d_{e}$ and $f_{e}$ denote the position error and contact force relative to the end-effector, respectively, and ε>0 is the stiffness coefficient. Equation (22) is applicable to a predetermined contact surface, which may be flat or irregularly curved. As such, the diagonal matrix Γ is customized to suit the specific structure and curvature of the surface. For example, when the contact force is aligned with the *z*-axis of the end-effector, Γ can be decomposed as Γ=diag(0,0,1).

Similarly, the motion tracking error is defined as

(23) $$\begin{array}{c} \chi_{e}=\overset{¯}{\Gamma }d_{e} \end{array}$$

where $\overset{¯}{\Gamma }=I-\Gamma =\mathrm{diag}\left(1,1,0\right)$ while 1 and 0 indicate the directions along which the manipulator can and cannot move, respectively.

Further, contact force (22) and tracking error (23) in the end-effector’s coordinate system are then converted into contact force and tracking error relative to the base coordinate system, denoted as $f_{b}$ and $\chi_{b}$ as follows:

(24) $$\begin{array}{c} \left\{\begin{array}{c} f_{b}=\varepsilon R^{T}\Gamma Rd_{b}\\ \chi_{b}=R^{T}\overset{¯}{\Gamma }Rd_{b} \end{array}\right. \end{array}$$

where $f_{b}=R^{T}f_{e}$, $\chi_{b}=R^{T}\chi_{e}$, and $d_{b}=R^{T}d_{e}$. The position error in Cartesian space is $d_{b}=r_{d}-r_{a}$.

Then, (24) is further simplified and rewritten as

(25) $$\begin{array}{c} Hd_{b}=n \end{array}$$

where $H=\left[\varepsilon R^{T}\Gamma R;R^{T}\overset{¯}{\Gamma }R\right]$, $n={\left[f_{b}^{T},\chi_{b}^{T}\right]}^{T}$, $n_{d}={\left[f_{d},0\right]}^{T}$. The control objective is to maintain a constant contact force at the desired value $f_{d}$ and ensure that $\chi_{b}$ converges to zero, i.e., $f_{b}\to f_{d}$ and $\chi_{b}\to 0$. This can be achieved by adjusting the joint angles to satisfy $n\to n_{d}$.

Define the error function $\upsilon =n_{d}-n=\left[f_{d}-f_{b};\chi_{b}\right]$, which can be further written as $\upsilon =n_{d}-Hd_{b}=n_{d}-H\left(r_{d}-r_{a}\right)$ from (25). Derivation of both sides of the equation yields

(26) $$\begin{array}{c} \dot{\upsilon }={\dot{n}}_{d}-H\left({\dot{r}}_{d}-J\dot{q}\right) \end{array}$$

To ensure that $\dot{\upsilon }$ converges to zero, a neural dynamic model $\dot{\upsilon }=-k\upsilon$ is designed with the constant k>0 [39], which finally yields

(27) $$\begin{array}{c} HJ\dot{q}=-{\dot{n}}_{d}+H\left({\dot{r}}_{d}+k\left(r_{d}-r_{a}\right)\right)-kn_{d} \end{array}$$

In the motion–force control criterion (27), both trajectory tracking and force control of the robotic manipulators are achieved simultaneously, as indicated by the inclusion of trajectory tracking control (3).

### 3.2. The Improved JDF Criterion

JDF schemes are crucial for eliminating joint drift, especially in RMP tasks, by ensuring accurate joint positions and preventing performance degradation or damage. Conventional JDF schemes use a performance index $d^{T}\dot{q}$, where $d=k\left(q-q_{0}\right)$ with k>0, and $q_{0}$ represents the initial joint state. However, decoupling joint drift in joint space from position errors in task space remains challenging. Geometrically, the improved JDF criterion achieves this decoupling by projecting corrective joint motions along the null space of the task Jacobian, thereby eliminating drift without affecting end-effector movement. Thus, this paper introduces an improved JDF criterion, denoted as

(28) $$\begin{array}{c} {\left(\lambda \left(I-J^{†}J\right)\left(q-q_{0}\right)\right)}^{T}\dot{q} \end{array}$$

where, λ>0 is used to adjust the response amplitude of the joint displacement, I denotes the identity matrix, $I-J^{†}J$ is the orthogonal projection matrix of J which leads to $J(I-J^{†}J)=0$.

**Lemma** **1.** *In the JDF criterion (28), the error vector $\nu =q-q_{0}\in R^{n}$ globally converges to zero, which means $\lim_{t\to \infty }q=q_{0}$, and the joint velocities globally converge to zero, i.e., $\lim_{t\to \infty }\dot{\nu }=0$.*

**Proof.**  Design the Lyapunov function as

(29) $$\begin{array}{c} V_{1}=\nu^{T}\nu /2 \end{array}$$

The derivative of (29) yields

(30) $$\begin{array}{c} \begin{array}{cc} {\dot{V}}_{1} & =\nu^{T}\dot{\nu }\\ \; & =\nu^{T}\left(-\lambda \left(I-J^{†}J\right)\left(q-q_{0}\right)\right)\\ \; & =\nu^{T}\left(-\lambda \left(I-J^{†}J\right)\nu \right)\\ \; & =-\lambda \nu^{T}\left(I-J^{†}J\right)\nu . \end{array} \end{array}$$

Then, using singular value decomposition matrix J yields

(31) $$\begin{array}{c} J=P\Lambda Q \end{array}$$

where $J\in R^{m\times n}$, $P\in R^{m\times m}$, $\Lambda \in R^{m\times n}$, $Q\in R^{n\times n}$, $P^{T}P=I$, and $Q^{T}Q=I$. It can be obtained that $J^{T}=Q^{T}\Lambda^{T}P^{T}$, and further gives

(32) $$\begin{array}{c} JJ^{T}=P\Lambda QQ^{T}\Lambda^{T}P^{T}=P\Lambda^{2}P^{T} \end{array}$$

The inverse of (32) gives

(33) $$\begin{array}{c} {\left(JJ^{T}\right)}^{-1}={\left(P^{T}\right)}^{-1}{\left(\Lambda^{2}\right)}^{-1}P^{-1}=P\Lambda^{-2}P^{T} \end{array}$$

which gives

(34) $$\begin{array}{cc} J^{†} & =J^{T}{\left(JJ^{T}\right)}^{-1}\\ \; & =Q^{T}\Lambda^{T}P^{T}\left(P\Lambda^{-2}P^{T}\right)=Q^{T}\Lambda^{T}\Lambda^{-2}P^{T} \end{array}$$

Set $\Lambda =\left[\begin{array}{cc} \Lambda_{a} & 0 \end{array}\right]$ with $\Lambda_{a}$ being the diagonal matrix and (34) is further reorganized as

(35) $$\begin{array}{c} J^{†}=Q^{T}\left[\begin{array}{c} \Lambda_{a}\\ 0 \end{array}\right]\Lambda^{-2}P^{T} \end{array}$$

which gives

(36) $$\begin{array}{c} J^{†}J=Q^{T}\left[\begin{array}{c} \Lambda_{a}\\ 0 \end{array}\right]\Lambda^{-2}P^{T}P\Lambda Q \end{array}$$

where $JJ^{T}$ is positive definite, and then

(37) $$\begin{array}{c} I-J^{†}J=\left[\begin{array}{cc} 0 & 0\\ 0 & I \end{array}\right]⪰0 \end{array}$$

where the symbol ⪰ indicates that each element in the preceding matrix is greater than or equal to the corresponding element in the following matrix.

Design a dynamic system as

(38) $$\begin{array}{c} \dot{q}=-\lambda \left(I-J^{†}J\right)\left(q-q_{0}\right). \end{array}$$

It is inferred that the dynamic system (38) converges to zero globally, which in turn leads to $\lim_{t\to \infty }\nu_{n-m}\left(t\right)=0$, $\lim_{t\to \infty }q_{n-m}\left(t\right)=q_{n-m}\left(0\right)$, where the associated eigenvalue of the subsystems is 1. Considering the equality constraint in (28) with $q_{n-m}\left(T_{a}\right)=q_{n-m}\left(0\right)$, where $T_{a}$ denotes the task duration, where the forward kinematic problem degenerates to the non-redundant case when $q_{n-m}\left(T_{a}\right)$ is fixed to $q_{n-m}\left(0\right)$, and thus $q_{m}\left(T_{a}\right)\in R^{m}$ such that $q_{m}\left(T_{a}\right)=q_{m}\left(0\right)$. This implies that the error ν converges globally to zero, i.e., $\lim_{t\to \infty }\nu \left(t\right)=0$. Furthermore, it is easy to obtain that the limit of $\dot{\nu }=\dot{q}=-\lambda \left(I-J^{†}J\right)\nu$ is $\lim_{t\to \infty }\dot{\nu }\left(t\right)=0$, i.e., the joint velocity converges to zero globally.

The proof is thus complete. □

**Property** **1** ([40])**.** *The improved JDF criterion (28) achieves complete decoupling between joint drift in joint space and position error in task space.*

**Proof.**  Before supplying it, design an error function as $\omega ={∥r_{d}-r_{a}∥}_{2}^{2}/2$, and then the evolution direction for the minimum ω is obtained as $-\zeta \partial \left(\omega \right)/\partial \left(q\right)=\zeta J^{T}\left(r_{d}-r_{a}\right)$, with ζ>0 by the gradient descent method.

Then, by integrating the dynamic system (38) as joint velocity compensation in $\dot{q}$, the calculation scheme is adjusted to fulfill the demands of repetitive motion. Consequently, it becomes attainable to accomplish

(39) $$\begin{array}{cc} \dot{q}=\zeta J^{T}\left(r_{d}-r_{a}\right) & +J^{†}{\dot{r}}_{d}-\lambda \left(I-J^{†}J\right)\left(q-q_{0}\right). \end{array}$$

Thus, based on the position error function in Cartesian space, $D=r_{d}-r_{a}$, differentiating it yields the derivative form $\dot{D}={\dot{r}}_{d}-{\dot{r}}_{a}={\dot{r}}_{d}-J\dot{q}$. Subsequently, substituting (39) further yields

(40) $$\begin{array}{c} \begin{array}{cc} \dot{D}= & {\dot{r}}_{d}-J\left(\zeta J^{T}D+J^{†}{\dot{r}}_{d}-\lambda \left(I-J^{†}J\right)\left(q-q_{0}\right)\right)\\ = & {\dot{r}}_{d}-\zeta JJ^{T}D-JJ^{†}{\dot{r}}_{d}+\lambda J\left(I-J^{†}J\right)\left(q-q_{0}\right)\\ = & -\zeta JJ^{T}D. \end{array} \end{array}$$

Define $\tau_{min}$ be the minimum eigenvalue of $JJ^{T}$, we get $-\zeta JJ^{T}D\leq -\zeta \tau_{\min}D$, i.e., $\dot{D}\leq -\zeta \tau_{\min}D$. Designing the Lyapunov candidate as $V=D^{T}D/2$ and taking its derivative yields $\dot{V}=D^{T}\dot{D}\leq -\zeta \tau_{\min}D^{T}D\leq 0$. According to Lyapunov theory, the position error D converges exponentially and globally to zero.

Hereto, $D_{i}\left(0\right)$ and $D_{i}$ is set to be the *i*-th element of D(0) and D, respectively. It can be deduced from (41) that $D_{i}\leq D_{i}\left(0\right)\exp\left(-\zeta \tau_{\min}t\right)$, and the right-hand term of the inequality is almost zero, which in turn leads to $D_{i}\to 0$.

The proof is thus complete. □

**Remark** **3.** *Leveraging Lemma 1 and Property 1, it’s evident that when a cyclic task is completed using the JDF criterion (28), the joint angles return to their initial state smoothly. This criterion effectively remedies joint angle drift phenomenon while enhancing control robustness and accuracy by completely decoupling it from end-effector position deviation, avoiding any mutual interference.*

### 3.3. The LMFC Scheme

This paper proposes the LMFC scheme, motivated by bioinspired principles of adaptive and compliant sensorimotor coordination, to address challenges such as incomplete kinematic information and multiple practical constraints, ensuring robust, multi-constraint management for accurate and safe manipulation in complex environments. It integrates physical limitations (12), obstacle avoidance (16), and end-effector orientation maintenance (21) into the motion–force control (27) and enhanced JDF criterion (28), reformulating them as a cohesive multi-objective TVQP framework. The resulting formulation yields

(41) minq˙Tq˙/2+λI−J†J(q−q0)Tq˙s.t.HJ^q˙=−n˙d+H(r˙d+k(rd−ra))−kndMq˙=0JBq˙⩽μq˙(t)∈Θ∥q¨∥∞≤ηmax

where J^ is used in place of J in the motion–force control criterion (27) to account for incomplete or implicit structural parameters of the manipulator.

**Remark** **4.** *The LMFC scheme, integrated within the TVQP framework (41), combines optimization and motion–force control metrics to satisfy multiple constraints, relying on the compatibility of four key criteria: physical constraints, obstacle avoidance, end-effector orientation maintenance, and joint angle drift elimination. The specific functional roles and contributions of these criteria are summarized in Table 2. By adjusting constraint proportions to meet task requirements and relaxing less critical constraints, the scheme enhances efficiency and adaptability, significantly improving task execution across diverse environments.*

Thus, the LMFC scheme (41), motivated by bioinspired principles of adaptive and compliant coordination, is ideally suited for redundant robotic manipulators, leveraging additional degrees of freedom to enhance flexibility, optimize task performance, navigate obstacles, maintain end-effector orientation, and efficiently distribute forces, thereby improving overall system robustness, precision, and versatility.

## 4. Neural Network Design

An RNN-based controller, motivated by bioinspired principles of adaptive sensorimotor learning, is developed to address redundancy in the LMFC framework by managing incomplete kinematic information and practical constraints, thereby ensuring robust, stable, and compliant performance in complex environments.

### 4.1. RNN-Based Controller

This section addresses various constraint limitations, including physical constraints, obstacle avoidance, end-effector orientation maintenance, and the JDF criterion, while enforcing physical constraints at the joint acceleration level through the proposed initial RNN-based controller. First, a non-negative relaxation term $g^{2}$ is introduced in optimization with inequality constraints as follows

(42) $$\begin{array}{c} J_{B}\dot{q}-\mu +g^{2}=0 \end{array}$$

where $g^{2}=g⋄g⪰0$.

Then, a Lagrangian function is established as

(43) $$\begin{array}{cc} L={\dot{q}}^{T}\dot{q}+D^{T}\dot{q}+\kappa_{1}^{T}(HJ\dot{q} & -N)+\kappa_{2}^{T}M\dot{q}\\ \; & +\kappa_{3}^{T}\left(J_{B}\dot{q}-\mu +g^{2}\right) \end{array}$$

where $D=\lambda \left(I-J^{†}J\right)\left(q-q_{0}\right)$ and $N=-{\dot{n}}_{d}+H\left({\dot{r}}_{d}+k\left(r_{d}-r_{a}\right)\right)-kn_{d}$ for presentation convenience, $\kappa_{1},\kappa_{2},\kappa_{3}$ are Lagrangian multipliers corresponding to constraints in (41).

According to the KKT condition [41], take partial derivative of (43) with respect to $\dot{q}$, $\kappa_{1}$, $\kappa_{2}$ and $\kappa_{3}$, which should eventually satisfy the following equation as

(44) $$\begin{array}{cc} \frac{\partial L}{\partial \dot{q}} & =\dot{q}+D+J^{T}H^{T}\kappa_{1}+M^{T}\kappa_{2}+J_{B}^{T}\kappa_{3}=0\\ \frac{\partial L}{\partial \kappa_{1}} & =HJ\dot{q}-N=0\\ \frac{\partial L}{\partial \kappa_{2}} & =M\dot{q}=0\\ \frac{\partial L}{\partial \kappa_{3}} & =J_{B}\dot{q}-\mu +g^{2}=0 \end{array}$$

Besides, the physical constraint is reconstructed using projection operator as a projection function to ensure the constraint of joint velocity, which is expressed as

(45) $$\begin{array}{cc} \dot{q} & =P_{\Theta }\left(\dot{q}-\frac{\partial L}{\partial \dot{q}}\right)\\ \; & =P_{\Theta }\left(-D-J^{T}H^{T}\kappa_{1}-M^{T}\kappa_{2}-J_{B}^{T}\kappa_{3}\right) \end{array}$$

where $P_{\Theta }\left(x\right)$ is defined as $P_{\Theta }\left(x\right)={\mathrm{argmin}}_{y\in \Theta }{∥x-y∥}_{2}$.

Thus, by utilizing (44) and (45) and extending the RNN controller [23] in its ordinary differential form, we obtain the following derived expression

(46) $$\begin{array}{cc} \ddot{q} & =\delta \left(-\dot{q}+P_{\Theta }\left(W\right)\right)\\ {\dot{\kappa }}_{1} & =\delta (HJ\dot{q}-N)\\ {\dot{\kappa }}_{2} & =\delta M\dot{q}\\ {\dot{\kappa }}_{3} & =\delta \left(J_{B}\dot{q}-\mu +g^{2}\right) \end{array}$$

where $W=-D-J^{T}H^{T}\kappa_{1}-M^{T}\kappa_{2}-J_{B}^{T}\kappa_{3}$ for presentation convenience, and δ>0∈R is used to adjust the convergence rate.

The neural dynamics $\ddot{q}$ described in Equation (46), generated by each iteration of the control process, ultimately gives rise to the control signal $\dot{q}$. The infinite norm of joint acceleration, denoted as a bound $\eta_{\max}$, is further transformed through the RNN controller (46) as follows:

(47) $$\begin{array}{c} {∥\delta \left(-\dot{q}+P_{\Theta }\left(W\right)\right)∥}_{\infty }\leq \eta_{\max}. \end{array}$$

Set Υ>0∈R to scale the acceleration level constraint, incorporating (47) into the controller (46), which yields

(48) $$\begin{array}{c} \Upsilon =\frac{{∥\delta \left(-\dot{q}+P_{\Theta }\left(W\right)\right)∥}_{\infty }}{{∥P_{\Upsilon }\left(\delta \left(-\dot{q}+P_{\Theta }\left(W\right)\right)\right)∥}_{\infty }} \end{array}$$

where $P_{\Upsilon }$ denotes projection operation on $\left\{x|∥x{∥}_{\infty }<\eta_{\max}\right\}$ thereby making the maximum absolute value less than $\eta_{\max}$. Hence, according to (48), the parameter Υ can be further equivalently reorganized as

(49) $$\begin{array}{c} \Upsilon =\left\{\begin{array}{c} 1,\;\mathrm{if}\;\delta \left(-\dot{q}+P_{\Theta }\left(W\right)\right)\in \left\{x|{∥x∥}_{\infty }<\eta_{\max}\right\}\\ \frac{{∥\delta \left(-\dot{q}+P_{\Theta }\left(W\right)\right)∥}_{\infty }}{\eta_{\max}}>1,\;\mathrm{otherwise}.\; \end{array}\right. \end{array}$$

Furthermore, the joint acceleration constraint (48) and the RNN controller (46) are combined and reorganized as

(50) $$\begin{array}{c} \begin{array}{cc} \Upsilon & =\frac{{∥\delta \left(-\dot{q}+P_{\Theta }\left(W\right)\right)∥}_{\infty }}{{∥P_{\Upsilon }\left(\delta \left(-\dot{q}+P_{\Theta }\left(W\right)\right)\right)∥}_{\infty }}\\ \ddot{q} & =\frac{\delta }{\Upsilon }\left(-\dot{q}+P_{\Theta }\left(W\right)\right)\\ {\dot{\kappa }}_{1} & =\frac{\delta }{\Upsilon }\left(HJ\dot{q}-N\right)\\ {\dot{\kappa }}_{2} & =\frac{\delta }{\Upsilon }M\dot{q}\\ {\dot{\kappa }}_{3} & =\frac{\delta }{\Upsilon }\left(J_{B}\dot{q}-\mu +g^{2}\right) \end{array} \end{array}$$

Compared to (46), (50) introduces just one additional computational procedure without significantly increasing complexity. The RNN controller (50) assumes prior knowledge of the Jacobian matrix J and includes joint angle acceleration constraints to keep accelerations within a specified range. When accelerations are within the range, Υ=1, indicating that $∥\ddot{q}{∥}_{\infty }<\eta_{\max}$. If accelerations exceed the constraint, $∥\ddot{q}{∥}_{\infty }=\eta_{\max}$, the convergence coefficient in (50) decreases to δ/Υ<δ, resulting in slower convergence. Consequently, rapid changes in joint angles and velocities decrease, fulfilling the imposed constraints on joint acceleration.

### 4.2. Reconstruction of RNN for Incomplete Kinematics

To address incomplete kinematic information, this section enhances the controller (50) by incorporating a learning law (7) for online estimation of the Jacobian matrix. This refinement results in a reconfigured controller that leverages the redundancy in the LMFC scheme by dynamically integrating learning and control, enabling real-time kinematic adaptation. Motivated by bioinspired principles of adaptive sensorimotor coordination, the resulting RNN-based controller is designed to be

(51) Υ=δ−q˙+PΘW^∞PΥδ−q˙+PΘW^∞q¨=δΥ−q˙+PΘW^κ˙1=δΥ(HJ^q˙−N)κ˙2=δΥMq˙κ˙3=δΥJBq˙−μ+g2q˙¯=q˙−c,with∥c∥≤c0ιJ^˙=r˙a−J^q˙¯q˙¯T

where W^=−D−J^THTκ1−MTκ2−JBTκ3, and c with $c_{0}>0$ is independent identically distributed noise introduced to enrich control signal diversity. Here, $\dot{q}$ denotes the nominal joint velocity used for standard feedback and control computations, whereas $\bar{\dot{q}}=\dot{q}-c$ represents a deliberately perturbed velocity employed exclusively for robust online Jacobian estimation, ensuring persistent excitation while preserving controller stability.

**Figure 2:**
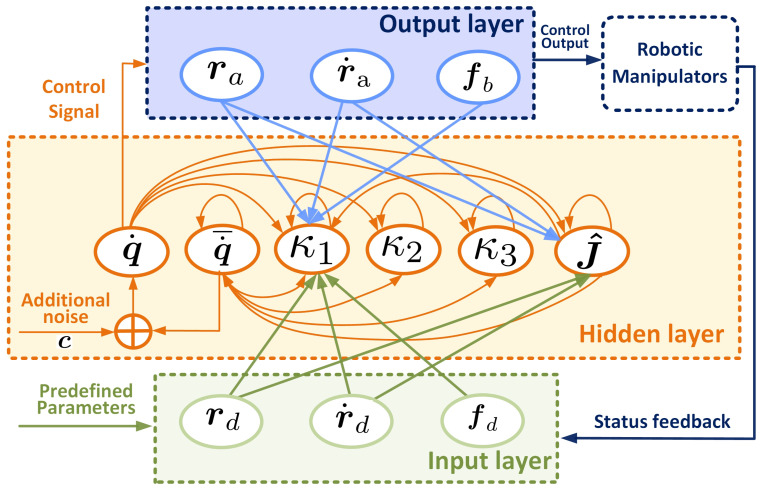
The architecture block diagram of RNN controller.

**Remark** **5.** *Figure 2 presents the block diagram of the reconstructed RNN controller (51), which adaptively estimates incomplete kinematic parameters using real-time measurements of end-effector and joint velocities, thereby seamlessly integrating learning and control within the loop. It should be noted that the proposed RNN is not a trained deep recurrent neural network (e.g., LSTM or GRU), but a continuous-time neural-dynamic optimization solver, representing a classical model-based method for online iterative computation rather than data-driven learning. This controller effectively handles acceleration-level constraints and unknown kinematics, with its recurrent structure providing history-aware updates and rapid online adaptation, thereby outperforming existing SOTA methods [8,10,15,34] in dynamic and uncertain environments.*

Nevertheless, real-world robotic systems face challenges including communication delays, sensor–actuator mismatches, and actuator-limited position accuracy. Drawing inspiration from biological sensorimotor systems, the LMFC framework integrates real-time delay compensation, predictive control, and robust design, with its bioinspired RNN-based controller dynamically coupling feedback and learning-based adaptation to achieve dexterous, compliant, and robust handling of disturbances, incomplete kinematics, and actuator uncertainties. The Jacobian learning law is seamlessly integrated with constraint-aware, online hybrid motion–force control and acceleration- and velocity-level TVQP handling, with identifiability ensured via manipulator redundancy and task diversity, sensor noise mitigated through tunable learning rates and low-pass filtering, and the perturbation c safely bounded and optimized ($c_{0}$) to enrich input signal diversity and facilitate exploration around saddle points. These biologically inspired mechanisms collectively confer strong robustness, enabling simultaneous satisfaction of multi-level constraints and continuous estimation of uncertain kinematics, while dynamic update adjustments maintain stable convergence even under slow or localized motion, making the framework suitable for complex industrial applications.

### 4.3. Theoretical Analysis

#### 4.3.1. Notations

To enhance readability, the following notations are collected at the beginning of the theoretical results:

J: The actual Jacobian matrix.

J^: The estimated Jacobian matrix.

J˜=J−J^: The estimation error of the Jacobian matrix.

r: The actual end-effector trajectory.

q: The joint angle vector.

$\dot{q}$: The joint angular velocity vector.

$\ddot{q}$: The joint angular acceleration vector.

$\bar{\dot{q}}$: The joint angular velocity used within the RNN controller (51), which is deliberately perturbed by the bounded noise c to enable persistent excitation for robust online Jacobian estimation.

c: An independent and identically distributed noise term, introduced to enrich and diversify the control system signals while preserving overall controller stability.

σ: The bound of the noise c.

Σ: A matrix defined as ${\overset{˜}{J}}^{T}\overset{˜}{J}\left(\bar{\dot{q}}+c\right){\left(\bar{\dot{q}}+c\right)}^{T}$.

$V_{2}$: The Lyapunov function associated with the Jacobian estimation error $\overset{˜}{J}$.

ι: The iteration step size used for learning in the RNN controller (51).

E: The expected value operator.

$\eta_{\max}$: The maximum joint angular acceleration bound.

w: A point within the convex set Θ.

$P_{\Theta }\left(w\right)$: The projection of w onto the convex set Θ.

e: An element within the convex set Θ.

W: Defined as $W=-D-J^{T}H^{T}\kappa_{1}-M^{T}\kappa 2-JB^{T}\kappa_{3}$ for the sake of presentation clarity.

Υ>0∈R: A positive scalar calculated using Equation (49) to scale the acceleration level constraint. Specifically, Υ=1 denotes that the constraint is satisfied.

δ>0∈R: A design parameter used to regulate the convergence rate.

$\kappa_{1},\kappa_{2},\kappa_{3}$: The Lagrangian multipliers corresponding to the constraints in the LMFC scheme (41).

$V_{3}$: The Lyapunov function associated with the new variable $d_{n}={\left[{\dot{q}}^{T},\kappa_{1}^{T},\kappa_{2}^{T},\kappa_{3}^{T}\right]}^{T}$.

#### 4.3.2. Learning Performance of the RNN Controller

The learning convergence of RNN controller (51) will be verified through theoretical analysis.

**Theorem** **1** (learning convergence)**.** *When using RNN controller (51) to solve the LMFC scheme (41), the estimation error J˜=J−J^ converges to zero.*

**Proof.**  Design a Lyapunov function as $V_{2}={∥\overset{˜}{J}∥}_{F}^{2}=\mathrm{tr}\left({\overset{˜}{J}}^{T}\overset{˜}{J}\right)/2$. Then, the derivation of $V_{2}$ yields

(52) V˙2=trJ˜˙J˜T=−tr1/ιJ˜T(r˙−J^q˙)q˙T=−tr1/ιΣ=−1/ι∥J˜(q˙¯+c)∥F2

where $\Sigma ={\overset{˜}{J}}^{T}\overset{˜}{J}\left(\bar{\dot{q}}+c\right){\left(\bar{\dot{q}}+c\right)}^{T}$, ${\dot{V}}_{2}\leq 0$ and $V_{2}>0$. From the LaSalle’s invariant set principle, it can be deduced that when ${\dot{V}}_{2}=0$, $\overset{˜}{J}\left(\bar{\dot{q}}+c\right)=0$ as t→∞.

Further multiply ${\left(\bar{\dot{q}}+c\right)}^{T}{\overset{˜}{J}}^{T}$ and calculate expected value as

(53) $$\begin{array}{cc} E & \left({\left(\bar{\dot{q}}+c\right)}^{T}{\overset{˜}{J}}^{T}\overset{˜}{J}\left(\bar{\dot{q}}+c\right)\right)\\ \; & =E\left(\mathrm{tr}\left(\Sigma \right)\right)+\mathrm{tr}\left(E\left({\overset{˜}{J}}^{T}\overset{˜}{J}\bar{\dot{q}}c^{T}\right)\right)+\mathrm{tr}\left(E\left({\overset{˜}{J}}^{T}\overset{˜}{J}c{\bar{\dot{q}}}^{T}\right)\right)\\ \; & =\mathrm{tr}\left(E\left({\overset{˜}{J}}^{T}\overset{˜}{J}\bar{\dot{q}}{\bar{\dot{q}}}^{T}\right)\right)+\mathrm{tr}\left(E\left({\overset{˜}{J}}^{T}\overset{˜}{J}\right)E\left(cc^{T}\right)\right)\\ \; & \;+\mathrm{tr}\left(E\left({\overset{˜}{J}}^{T}\overset{˜}{J}\bar{\dot{q}}\right)E^{T}\left(c\right)\right)+\mathrm{tr}\left(E\left({\bar{\dot{q}}}^{T}{\overset{˜}{J}}^{T}\overset{˜}{J}\right)E\left(c\right)\right)\\ \; & =\mathrm{tr}\left(E\left({\bar{\dot{q}}}^{T}{\overset{˜}{J}}^{T}\overset{˜}{J}\bar{\dot{q}}\right)\right)+\sigma^{2}\mathrm{tr}\left(E\left({\overset{˜}{J}}^{T}\overset{˜}{J}\right)\right)\\ \; & =0,\;\;\mathrm{as}\;t\to \infty \end{array}$$

where σ is the bound of the noise c.

Since $\mathrm{tr}\left(E\left({\bar{\dot{q}}}^{T}{\overset{˜}{J}}^{T}\overset{˜}{J}\bar{\dot{q}}\right)\right)\geq 0$, $\mathrm{tr}\left(E\left({\overset{˜}{J}}^{T}\overset{˜}{J}\right)\right)=E\left(\mathrm{tr}\left({\overset{˜}{J}}^{T}\overset{˜}{J}\right)\right)=E\left(∥\overset{˜}{J}{∥}_{F}^{2}\right)=0$, and thus J˜=J−J^=0 as t→∞. The proof is thus complete. □

**Lemma** **2.** *According to [42], for any convex set* **Θ** *, if w∈Θ, then*

(54) $$\begin{array}{c} {\left(w-P_{\Theta }\left(w\right)\right)}^{T}\left(P_{\Theta }\left(w\right)-e\right)\geq 0\;\forall e\in \Theta \end{array}$$

*which gives*

(55) $$\begin{array}{cc} \; & \;{\left(w-P_{\Theta }\left(w\right)\right)}^{T}\left(w-e\right)\\ \; & ={\left(w-P_{\Theta }\left(w\right)\right)}^{T}\left(w-P_{\Theta }\left(w\right)+P_{\Theta }\left(w\right)-e\right)\\ \; & =∥\left(w-P_{\Theta }\left(w\right)\right){∥}_{2}^{2}+{\left(w-P_{\Theta }\left(w\right)\right)}^{T}\left(P_{\Theta }\left(w\right)-e\right)\geq 0 \end{array}$$

The learning convergence of RNN controller (51) is theoretically guaranteed under the assumptions that (i) the manipulator dynamics are bounded and Lipschitz continuous, (ii) all acceleration- and velocity-level constraints are convex, (iii) joint velocity measurements are available with bounded noise, and (iv) system delays are sufficiently small relative to the controller update rate. Under these conditions, Lyapunov-based analysis ensures that the Jacobian estimation error J˜=J−J^ converges to zero (Theorem 1). Moreover, the RNN-based estimator dynamically compensates for small model mismatches and sensor noise, with the perturbation term c safely bounded and optimized to preserve convergence even under slow or localized motion, thereby providing robustness in practical scenarios.

#### 4.3.3. Control Performance of the RNN Controller

The control convergence of RNN controller (51) will be verified through theoretical analysis.

**Theorem** **2** (control convergence)**.** *The neural dynamics $d_{n}={\left[{\dot{q}}^{T},\kappa_{1}^{T},\kappa_{2}^{T},\kappa_{3}^{T}\right]}^{T}$ in the RNN controller (51) converges globally to the steady state while the joint acceleration constraint is satisfied.*

**Proof.**  The joint acceleration constraint is first proved. In view of the proof in Theorem 1, the estimated Jacobian matrix is denoted by J in the following proof.

When the joint acceleration does not satisfy the constraint, it is further derived from (49) and (51) that

(56) $$\begin{array}{c} \begin{array}{cc} ∥\ddot{q}{∥}_{\infty } & ={∥\frac{\delta }{\Upsilon }\left(-\dot{q}+P_{\Theta }\left(W\right)\right)∥}_{\infty }\\ \; & ={∥\frac{\eta_{\max}\delta \left(-\dot{q}+P_{\Theta }\left(W\right)\right)}{\delta \left(-\dot{q}+P_{\Theta }\left(W\right)\right)}∥}_{\infty }\\ \; & =\eta_{\max}{∥\frac{\delta \left(-\dot{q}+P_{\Theta }\left(W\right)\right)}{\delta \left(-\dot{q}+P_{\Theta }\left(W\right)\right)}∥}_{\infty }\\ \; & =\eta_{\max} \end{array} \end{array}$$

If the joint acceleration satisfies the predetermined constraint, i.e., Υ=1, we get

(57) $$\begin{array}{cc} ∥\ddot{q}{∥}_{\infty } & ={∥\frac{\delta }{\Upsilon }\left(-\dot{q}+P_{\Theta }\left(W\right)\right)∥}_{\infty }\\ \; & ={∥\delta \left(-\dot{q}+P_{\Theta }\left(W\right)\right)∥}_{\infty }\\ \; & <\eta_{\max}. \end{array}$$

Based on the above discussion, it can be inferred that $\ddot{q}\leq \eta_{\max}$ always holds, i.e., the joint acceleration satisfies the constraint.

Then, global convergence of the RNN controller (51) is proved. Define a new variable as $d_{n}={\left[{\dot{q}}^{T},\kappa_{1}^{T},\kappa_{2}^{T},\kappa_{3}^{T}\right]}^{T}$, and in turn the corresponding control law is reorganized as

(58) $$\begin{array}{c} \frac{\Upsilon }{\delta }{\dot{d}}_{n}=-d_{n}+P_{\overset{¯}{\Theta }}\left(d_{n}-h\left(d_{n}\right)\right) \end{array}$$

where $P_{\overset{¯}{\Theta }}=\left\{\dot{q},\kappa_{1},\kappa_{2},\kappa_{3}\right\}$, and h(·) denotes

(59) $$\begin{array}{cc} h(d_{n}) & =h(\dot{q},\kappa_{1},\kappa_{2},\kappa_{3})\\ \; & =\left[\begin{array}{c} \dot{q}+D+J^{T}H^{T}\kappa_{1}+M^{T}\kappa_{2}+J_{B}^{T}\kappa_{3}\\ -HJ\dot{q}+N\\ -M\dot{q}\\ \mu -g^{2}-J_{B}\dot{q} \end{array}\right] \end{array}$$

The gradient of $h(d_{n})$ is further obtained as

(60) $$\begin{array}{c} \nabla h=\frac{\partial h(d_{n})}{\partial d_{n}}=\left[\begin{array}{cccc} I & JH & M & J_{B}\\ -H^{T}J^{T} & 0 & 0 & 0\\ -M^{T} & 0 & 0 & 0\\ -J_{B}^{T} & 0 & 0 & 0 \end{array}\right] \end{array}$$

which gives

(61) $$\begin{array}{c} \nabla h+\nabla^{T}h=\left[\begin{array}{cccc} 2I & 0 & 0 & 0\\ 0 & 0 & 0 & 0\\ 0 & 0 & 0 & 0\\ 0 & 0 & 0 & 0 \end{array}\right]. \end{array}$$

It can be seen that Equation (61) is a semi-positive definite matrix. From the mean-value theorem, it follows that for any *a*, *b*, and *c*, when c=μa+(1−μ)b,(0≤μ≤1), there exists h(a)−h(b)=∇h(c)(a−b), and thus it further follows that

(62) $$\begin{array}{c} {\left(a-b\right)}^{T}\left(h\left(a\right)-h\left(b\right)\right)={\left(a-b\right)}^{T}\nabla h\left(c\right)\left(a-b\right)\geq 0. \end{array}$$

Equation (62) yields that h(·) is a monotonic function. Defining a Lyapunov function $V_{3}={∥d_{n}-P_{\overset{¯}{\Theta }}\left(d_{n}\right)∥}_{2}^{2}/2$, it can be obtained that $V_{3}\geq 0$ holds when $d_{n}\in \overset{¯}{\Theta }$, and thus the time derivative of $V_{3}$ is calculated as

(63) $$\begin{array}{c} \begin{array}{cc} \; & {\dot{V}}_{3}={\left(d_{n}-P_{\overset{¯}{\Theta }}\left(d_{n}\right)\right)}^{T}\dot{d_{n}}\\ \; & =-{\left(d_{n}-P_{\overset{¯}{\Theta }}\left(d_{n}\right)\right)}^{T}\left(d_{n}-P_{\overset{¯}{\Theta }}\left(d_{n}-h\left(d_{n}\right)\right)\right)\delta /\Upsilon \end{array} \end{array}$$

By Lemma 2 and monotony of h(·), it is deduced that ${\left(d_{n}-P_{\overset{¯}{\Theta }}\left(d_{n}\right)\right)}^{T}\left(d_{n}-P_{\overset{¯}{\Theta }}(d_{n}-\right.$

$\left.h(d_{n}))\right)\geq 0$. It is finally concluded that ${\dot{V}}_{3}\leq 0$ holds when $d_{n}\in \overset{¯}{\Theta }$, therefore, $d_{n}$ converges to $\overset{¯}{\Theta }$ according to the LaSalle’s invariance principle. The proof is thus complete. □

The control convergence of RNN controller (51) is analyzed under the same assumptions listed above: bounded and Lipschitz continuous manipulator dynamics, convex acceleration- and velocity-level constraints, bounded joint velocity measurement noise, and sufficiently small system delays. Within these conditions, the Lyapunov-based proof and TVQP framework guarantee that joint accelerations remain within the prescribed bounds, while the neural dynamics $d_{n}$ converge globally to the steady-state set $\overset{¯}{\Theta }$, as stated in Theorem 2. Robustness to small uncertainties, sensor noise, and bounded delays is ensured by the RNN-based adaptive estimator and the constraint-aware TVQP design.

## 5. Simulation and Physical Experiment

In this section, simulations demonstrate the practicality and effectiveness of our proposed scheme in achieving learning and control performance while satisfying multiple constraints. Physical experiments further validate the effectiveness of the scheme in real-world scenarios.

### 5.1. Simulation Illustration

Simulations in MATLAB (R2023a) and CoppeliaSim involve the manipulator performing trajectory tracking tasks for hybrid motion–force control in complex environments. The proposed scheme address multiple constraints and practical application requirements while verifying the performance of estimating incomplete kinematic information through the integration of learning and control components.

The parameter settings are as follows: The initial state setting of the joints is randomly set to $q\left(0\right)={\left[-0.2670,0.6269,-0.6976,1.0613,-1.5069,-2.2772\right]}^{T}$ rad, $\dot{q}\left(0\right)={\left[0,0,0,0,0,0\right]}^{T}$ rad/s, $\ddot{q}\left(0\right)={\left[0,0,0,0,0,0\right]}^{T}$ rad/s^2^. The error between the actual and the desired initial position is defined as $r_{a0}-r_{d0}=0.1$ m. The corresponding kinematic parameters for the assumed values of the actual Jacobian matrix are shown in Table 3. The joint angle, velocity and acceleration constraints are set to $q^{+}=-q^{-}={\left[\pi ,\pi ,\pi ,\pi ,\pi ,\pi \right]}^{T}$ rad, ${\dot{q}}^{+}=-{\dot{q}}^{-}={\left[0.5,0.5,0.5,0.5,0.5,0.5\right]}^{T}$ rad/s, ${\ddot{q}}^{+}=-{\ddot{q}}^{-}={\left[1,1,1,1,1,1\right]}^{T}$ rad/s^2^, respectively. Hence, $\eta_{\max}=1$ rad/s^2^. The task duration was set to 5 s. The maintained contact force $f_{d}$ is set to 0.5 N, and ε=100.

The RNN controller’s design parameter δ=1000 is chosen to guarantee global stability while maintaining adherence to practical constraints, thereby facilitating efficient convergence to the solution of the LMFC scheme. The initial values for the Lagrangian multipliers, $\kappa_{1}$, $\kappa_{2}$, and $\kappa_{3}$, are randomly generated within the range of 0 to 0.1. A non-negative relaxation term $g^{2}=10I\in R^{n}$ addresses inequality constraints, while the scalar Υ modulates acceleration levels based on δ, physical constraints, and joint motion. When accelerations remain within limits, Υ=1 ensures $∥\ddot{q}{∥}_{\infty }<\eta_{\max}$; if exceeded, the convergence coefficient decreases to δ/Υ<δ, slowing convergence and mitigating abrupt joint dynamics changes. The learning step sizes, ι, are set to $10^{-5}$ and $10^{-6}$ to assess the learning performance of the controller under different conditions. For obstacle avoidance, safety and activation thresholds, $s_{o}=0.05\;m$ and $s_{a}=0.10\;m$, respectively, trigger the avoidance mechanisms.

#### 5.1.1. Verification of Motion–Force Control Performance

**Figure 3:**
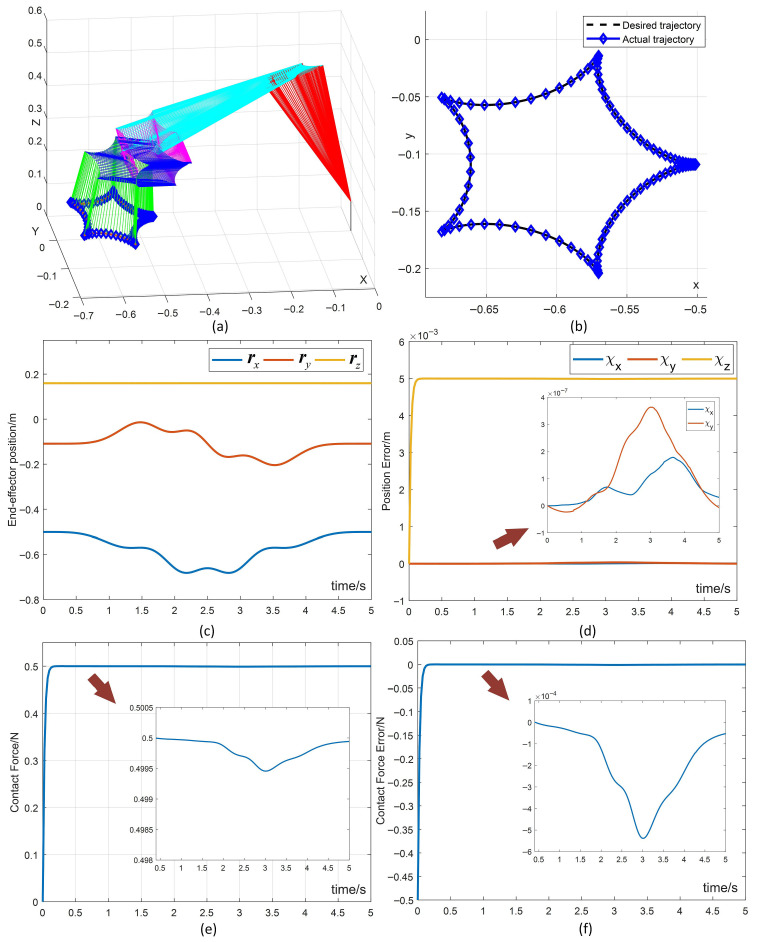
Simulation results of the proposed scheme for hybrid motion–force control in trajectory tracking tasks. (**a**) Motion of the manipulator. (**b**) Actual and desired trajectory of the end-effector. (**c**,**d**) End-effector position and corresponding errors. (**e**,**f**) Contact force and corresponding errors.

Figure 3 presents the simulation results of the proposed control scheme for motion–force hybrid control of the robotic manipulator with an unknown kinematic model, considering various constraint limitations during a tracking task. These simulation findings provide convincing evidence that supports the effectiveness of the proposed method. Specifically, Figure 3a displays the stable oscillations of the configuration of the manipulator during task execution. Figure 3b shows a close alignment between the actual trajectory and the desired trajectory throughout the task. Figure 3c,d illustrate the end-effector’s position and its corresponding position error, showcasing a smooth and stable operation. Specifically, the position errors along the *x*-axis and *y*-axis fluctuate within the tiny range of $10^{-7}$ m, while the *z*-axis position errors rapidly converge to the desired values, effectively maintaining the contact force along the *z*-axis at the desired level. Furthermore, in Figure 3e,f, the contact force and its corresponding errors along the *z*-axis direction of the end-effector are presented. It is evident that the contact force promptly converges to the desired value and eventually stabilizes around the target value, with the contact force error reaching a steady state of $10^{-4}$ N. The results confirm that the proposed control scheme effectively achieves high accuracy in controlling both the position and contact force of the end-effector. This confirms the effectiveness of the method in addressing the challenges of hybrid motion–force control during trajectory tracking tasks.

#### 5.1.2. Verification of the Physical Constraint

**Figure 4:**
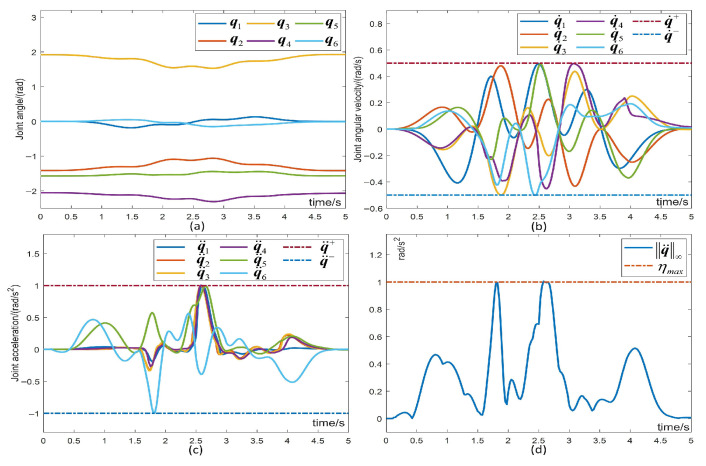
Joint angle, joint velocity and joint acceleration and the corresponding physical constraints.

To validate that the proposed scheme satisfies the physical constraints, Figure 4 presents the joint angles, velocities, and accelerations of the manipulator during the trajectory tracking task. Figure 4a illustrates that the joint angles fluctuate smoothly within the predetermined constraints throughout task execution, with the final joints returning to their initial state. Figure 4b demonstrates the joint velocity adhering to the specified constraint range. Figure 4c exemplifies the joint acceleration satisfying the physical constraint, confirming the robust performance of the scheme. Figure 4d depicts that the infinite norm of the joint acceleration is constrained during task execution, which is almost at the boundary, but well constrained. This comprehensive evaluation confirms the scheme’s effective fulfillment of physical constraints.

#### 5.1.3. Verification of the Obstacle Avoidance Constraint

**Figure 5:**
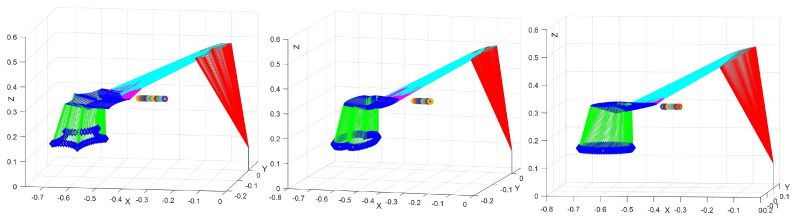
The performance of obstacle avoidance constraints in different trajectory tracking tasks for hybrid motion–force control.

In the simulation, obstacle points B represent simplified points of arbitrarily shaped obstacles closest to the manipulator, randomly positioned within a specific range, i.e., between (−0.4,−0.05,0.3) m and (−0.3,−0.05,0.3) m. The critical point A is defined as the nearest point on the link or its extension to the obstacle. The matrix $J_{B}$ is derived by transforming the Jacobian matrix $J_{A}$ of the critical point A and the vector ${\vec{p}}_{\mathrm{AB}}$. Figure 5 illustrates the obstacle avoidance performance of the proposed motion–force hybrid control in different trajectory tracking tasks. The configurations of the manipulator under obstacle points for various trajectories demonstrate its effective obstacle avoidance capabilities, maintaining a safe distance throughout. This confirms the effectiveness of the obstacle avoidance constraints.

#### 5.1.4. Verification of the End-Effector Orientation Maintaining Constraint

**Figure 6:**
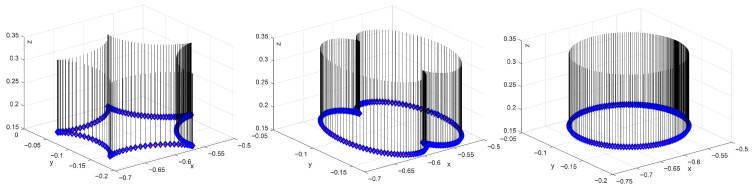
End-effector orientation maintenance in different trajectory tracking tasks for hybrid motion–force control.

**Figure 7:**
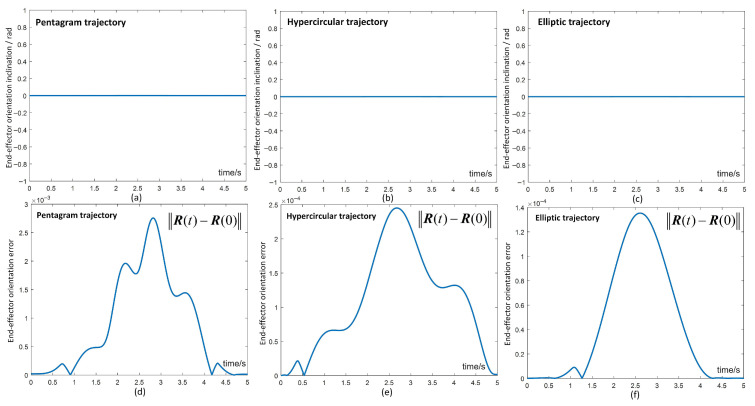
(**a**–**f**) The end-effector orientation inclination and corresponding errors in different trajectory tracking tasks for hybrid motion–force control.

To further validate that the scheme satisfies the end-effector orientation maintenance constraints, we assume that the end-effector maintains perpendicular to the task space throughout the trajectory tracking task. As illustrated in Figure 6, the scheme consistently preserves the end-effector orientation across a variety of trajectory tracking tasks. Quantitative analysis, as presented in Figure 7, provides insight into the orientation inclination and the corresponding errors of the end-effector with respect to the normal vector of the task plane during different trajectory tracking tasks. Additionally, when considering Figure 4 in conjunction with the provided data, it is evident that the manipulator consistently complies with the physical constraints while ensuring continuous maintenance of the end-effector orientation. Therefore, we can infer that this scheme is able to efficiently and consistently maintain the desired end-effector orientation.

#### 5.1.5. Verification of the Joint-Drift-Free Scheme

**Figure 8:**
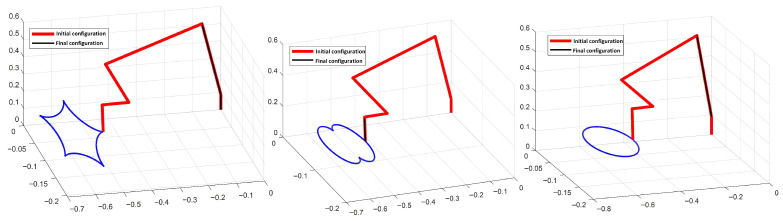
The performance of joint-drift-free scheme in different trajectory tracking tasks for hybrid motion–force control.

Figure 8 illustrates that, upon completion of various closed-loop trajectory tracking tasks, the joint angles return to their initial states, while Figure 4 shows that joint velocities and accelerations smoothly converge to zero. Table 4 provides a detailed comparison of the final versus initial joint angles when the JDF criterion (28) is applied or omitted during trajectory tracking. Geometrically, this behavior can be understood as the JDF criterion projecting corrective joint motions along the null space of the task Jacobian, thereby eliminating drift without affecting end-effector motion. The results clearly demonstrate that joint-angle drift is substantially reduced when the JDF criterion is applied. Overall, these simulations confirm the efficiency and practical utility of the improved JDF scheme in mitigating joint drift.

#### 5.1.6. Verification of Robustness of the RNN Controller

**Figure 9:**
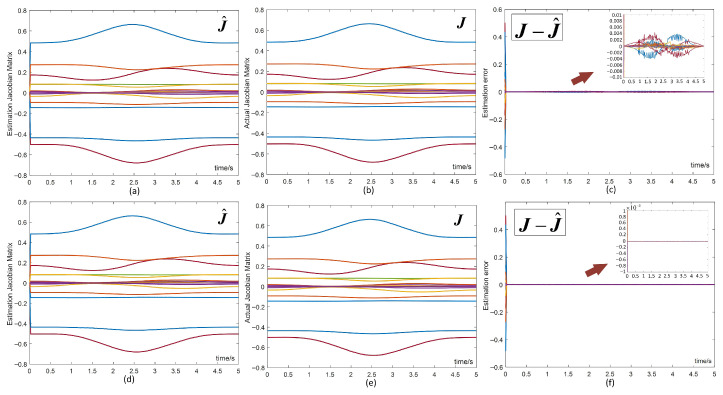
Learning performance of the RNN controller with different step sizes ι (**a**–**c**) $\iota =10^{-5}$, (**d**–**f**) $\iota =10^{-6}$.

To validate the learning performance, we assume that the kinematic parameters of the manipulator are unknown. The study presents the estimated Jacobian matrix, the assumed value of the actual Jacobian matrix, and the deviations between them. To assess the learning performance of the RNN controller at different step sizes, the design parameter ι is set to $10^{-5}$ and $10^{-6}$, respectively. Figure 9 illustrates the learning performance of the RNN controller for varying values of ι. Figure 9a–c show that when $\iota =10^{-5}$, the estimation error converges to a small value, fluctuating within $10^{-2}$. Figure 9d–f reveal that when $\iota =10^{-6}$, the error converges to an even smaller exponential level, yielding superior estimation performance. This confirms that the learning performance of the RNN controller improves as the step size ι decreases, enabling accurate estimation of the real Jacobian matrix. It is further concluded that the RNN controller is capable of learning kinematic uncertainties and external disturbances from real-time data, allowing it to estimate and compensate for kinematic accuracy uncertainties induced by actuators, thus ensuring stable performance even in systems with degraded actuator accuracy.

#### 5.1.7. Discussion of Comparison with Existing Methods

Across all simulation and experimental evaluations, LMFC exhibits clear and consistent advantages over existing methods. First, unlike motion-only controllers that neglect uncertain kinematics and multi-level constraints, LMFC simultaneously enforces physical limits, obstacle avoidance, orientation maintenance, and joint-drift suppression. This unified treatment yields position-tracking errors on the order of $10^{-7}\;m$ and force-tracking errors below $10^{-4}\;N$, substantially outperforming standard baselines [4,10,32,33]. Second, relative to prior hybrid motion–force schemes [13,15,34], the integrated RNN-based Jacobian estimator provides reliable online adaptation to unknown and time-varying kinematics, achieving Jacobian estimation errors as low as $10^{-6}$–$10^{-2}$. In contrast, conventional adaptive or model-based methods [4,7,13,16,17,20,32] exhibit significantly larger residual errors under the same conditions. Third, LMFC’s kinematic-level TVQP formulation—with acceleration-level constraints and velocity-level decision variables—enables stable optimization and effectively decouples joint drift from task-space regulation. This yields near-zero terminal joint velocities and accelerations and markedly reduces drift compared with JDF-free baselines [7,12,13,14,15,16,17,18,20,21,35].

Overall, these quantitative improvements—including tighter tracking, faster force convergence, reduced estimation error, and substantially enhanced constraint satisfaction—demonstrate that LMFC offers more precise, robust, and constraint-aware motion–force coordination than existing state-of-the-art controllers.

### 5.2. Implementation & Verification

To further evaluate and validate the practicality and feasibility of each constraint proposed in the LMFC scheme, as well as to examine the balance between these constraints, simulation demonstrations are conducted using the 7 DOF redundant manipulator, Franka Panda, in MATLAB and CoppeliaSim.

The parameter settings for the Franka Panda manipulator are as follows: The initial joint angles are randomly assigned, while the initial joint angular velocity and acceleration are set to ${\left[0,0,0,0,0,0,0\right]}^{T}\;\mathrm{rad}/s$ and ${\left[0,0,0,0,0,0,0\right]}^{T}\;\mathrm{rad}/s^{2}$, respectively. The kinematic parameters corresponding to the assumed actual Jacobian matrix values are listed in Table 5. The joint angle constraints are defined as $q^{-}=\left[-2.897,-1.763,-2.897,-3.072,-2.897,0.017,-2.897\right]$ and $q^{+}=\left[2.897,1.763,2.897,-0.070,2.897,3.752,2.897\right]$, while the joint angular velocity limits are ${\dot{q}}^{+}=-{\dot{q}}^{-}={\left[1.5,1.5,1.5,1.5,1.5,1.5,1.5\right]}^{T}\;\mathrm{rad}/s$. The initial design parameters $\kappa_{1},\kappa_{2},\kappa_{3}$ are randomly generated within [0,0.2]. The desired contact force $f_{d}$ and the parameter ε are set to 1N and 100, respectively. For the RNN controller, the design parameter δ is assigned a value of 1500. In the obstacle avoidance constraint, the safety threshold $s_{o}$ and activation threshold $s_{a}$ are set to 0.05m and 0.10m, respectively. Other parameters used in the demonstration are consistent with those employed in previous simulations.

**Figure 10:**
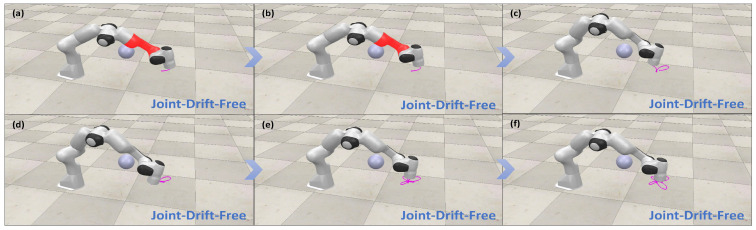
(**a**–**f**) Simulation demonstration of motion–force hybrid control for robotic manipulators with the joint-drift-free scheme.

**Figure 11:**
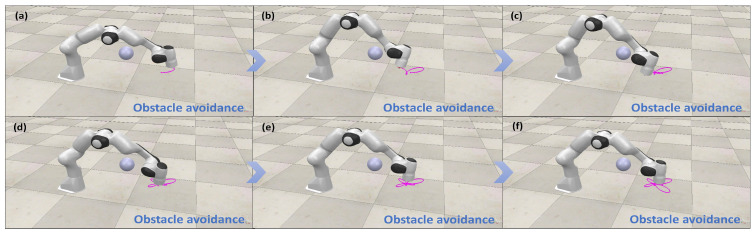
(**a**–**f**) Simulation demonstration of motion–force hybrid control for robotic manipulators with obstacle avoidance constraints.

**Figure 12:**
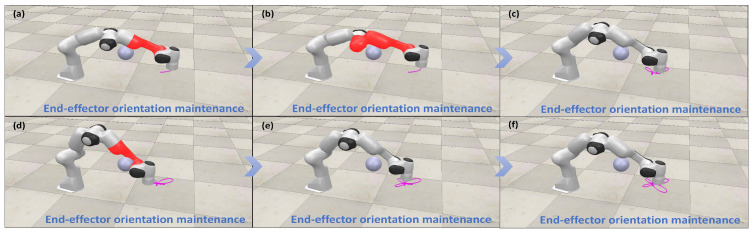
(**a**–**f**) Simulation demonstration of motion–force hybrid control for robotic manipulators with end-effector orientation maintaining constraints.

**Figure 13:**
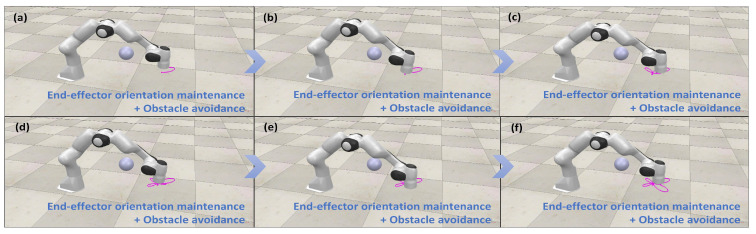
(**a**–**f**) Simulation demonstration of motion–force hybrid control for robotic manipulators with both obstacle avoidance and end-effector orientation maintaining constraints.

Figure 10, Figure 11, Figure 12 and Figure 13 present simulations of the motion–force hybrid control of the robot manipulator under three individual constraints and their combination, all while adhering to physical constraints: a joint drift-free scheme, an obstacle avoidance constraint, an end-effector orientation maintenance constraint, and a combined constraint of orientation maintenance and obstacle avoidance. The results indicate that the manipulator successfully executes the target task in each scenario. Figure 10 demonstrates that the enhanced JDF criterion effectively eliminates joint drift by smoothly restoring joint angles to their initial configurations during cyclic tasks, decoupling drift from end-effector deviations and bolstering control robustness. Figure 11 illustrates that the obstacle avoidance constraint takes advantage of redundancy, expanding the configuration space and allowing the LMFC scheme to plan optimal paths for safe and smooth navigation in cluttered environments. Figure 12 reveals that the end-effector orientation constraint makes use of redundant DOFs to maintain kinematic feasibility and ensure stable orientation within the redundant system. Moreover, Figure 13 shows that when both the end-effector orientation maintenance and obstacle avoidance constraints are applied concurrently, the manipulator successfully avoids obstacles while preserving the end-effector orientation. Additionally, the LMFC scheme efficiently distributes forces across redundant DOFs, enhancing stability and reducing task completion times, particularly in fine manipulation tasks, while ensuring the satisfaction of joint angle and joint velocity constraints.

**Figure 14:**
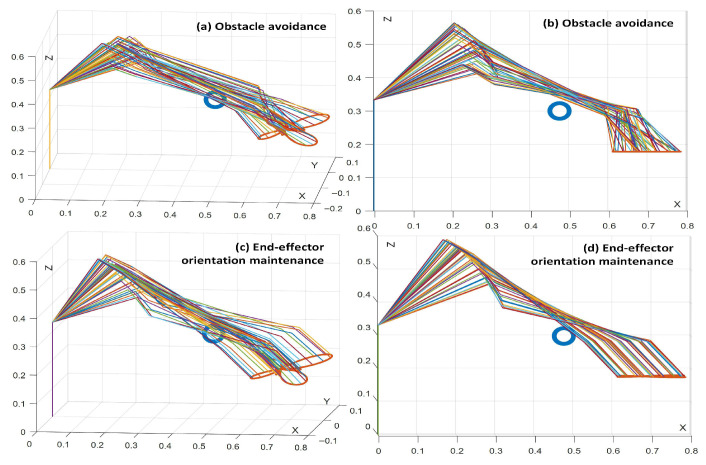
Motion configuration of robotic manipulators during the trajectory tracking task in motion–force hybrid control. (**a**,**b**) Obstacle avoidance constraints, (**c**,**d**) end-effector orientation maintaining constraints.

Figure 14 illustrates the manipulator’s motion configuration during a trajectory-tracking task under these combined constraints within the motion–force hybrid control framework. The results confirm that the obstacle avoidance constraint ensures safe navigation around obstacles, while the orientation maintenance constraint stabilizes the end-effector’s orientation. These findings validate the effectiveness of simultaneously applying both constraints, demonstrating their practical utility in enhancing the safety and stability of manipulator operations for specific task requirements.

**Figure 15:**
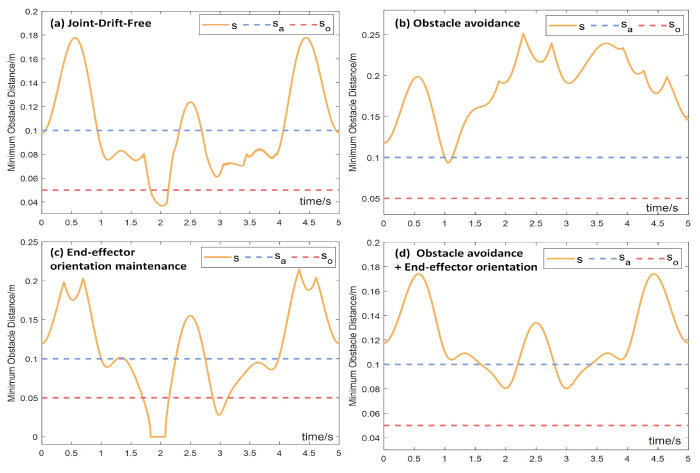
Relationship between the shortest distance *s*, safety distance $s_{o}$, and activation distance $s_{a}$ during the trajectory tracking task in motion–force hybrid control. (**a**) Joint-drift-free scheme, (**b**) obstacle avoidance constraints, (**c**) end-effector orientation maintaining constraints, (**d**) both obstacle avoidance and end-effector orientation maintaining constraints.

Figure 15 illustrates the relationship curves between the shortest distance *s* from the critical point A to the obstacle point B, the obstacle avoidance safety distance $s_{o}$, and the obstacle avoidance activation distance $s_{a}$ during the trajectory tracking task under the four aforementioned conditions in motion–force hybrid control. The quantitative minimum values of these parameters under different constraint configurations are summarized in Table 6. It can be observed that, under the obstacle avoidance constraint, the shortest distance *s* is maintained as large as possible. Once *s* falls below the activation distance $s_{a}$, the manipulator is promptly driven away from the obstacle, ensuring *s* remains above $s_{a}$. When both end-effector orientation maintenance and obstacle avoidance constraints are applied simultaneously, *s* consistently remains near the saddle point region of $s_{a}$ and always below the safety distance $s_{o}$, thereby effectively achieving the obstacle avoidance.

**Figure 16:**
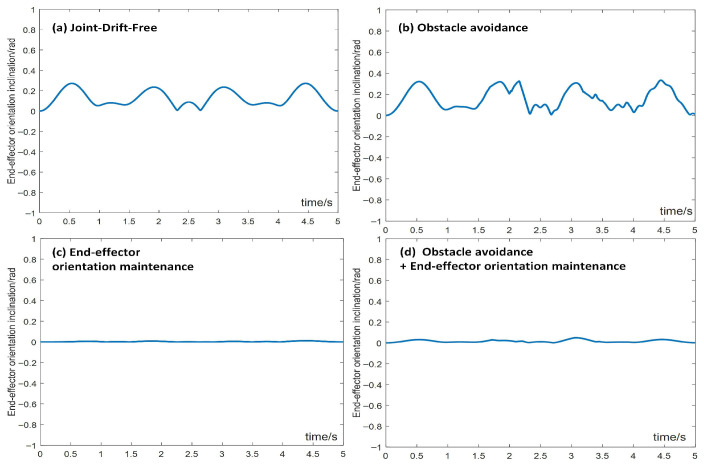
End-effector orientation inclination during the trajectory tracking task in motion–force hybrid control. (**a**) Joint-drift-free scheme, (**b**) obstacle avoidance constraints, (**c**) end-effector orientation maintaining constraints, (**d**) both obstacle avoidance and end-effector orientation maintaining constraints.

Figure 16 shows the end-effector orientation inclination of the manipulator during the trajectory tracking task under the aforementioned four conditions in motion–force hybrid control. Under the end-effector orientation maintenance constraint, the end-effector’s orientation remains perfectly stable in the desired posture throughout the task, enabling smooth and steady operation. When both end-effector orientation maintenance and obstacle avoidance constraints are considered, the end-effector’s orientation largely maintains the desired posture, ensuring stable operation while avoiding obstacles. Furthermore, under the joint-drift-free scheme, the end-effector’s orientation at task completion matches the initial orientation, effectively resolving joint angle drift in cyclic tasks.

**Figure 17:**
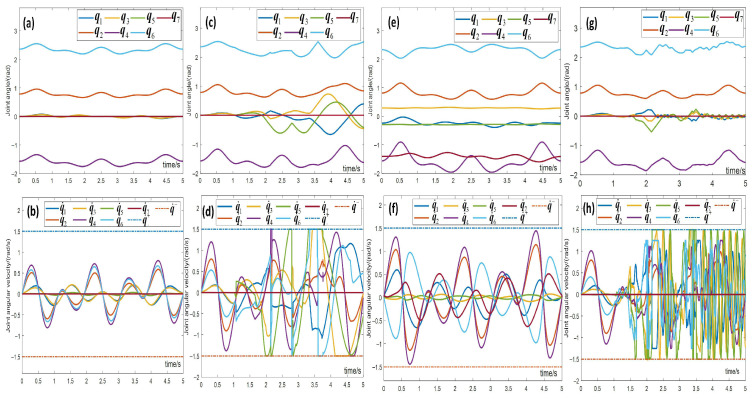
The joint angles and joint velocities during the trajectory tracking task in motion–force hybrid control. (**a**,**b**) Joint-drift-free scheme, (**c**,**d**) obstacle avoidance constraints, (**e**,**f**) end-effector orientation maintaining constraints, (**g**,**h**) both obstacle avoidance and end-effector orientation maintaining constraints.

Figure 17 displays the joint angles and joint angular velocities of the manipulator during the trajectory tracking task under the aforementioned four conditions in motion–force hybrid control. The joint angles and velocities remain well within the desired physical constraints under all conditions, demonstrating the effectiveness of physical constraints in ensuring smooth and stable joint operation. Under the Joint-Drift-Free scheme, the joint angles at task completion are consistent with the initial angles, and the joint angular velocities decrease steadily to zero, effectively addressing joint angle drift in cyclic tasks and enhancing manipulator safety. Additionally, under the separate and combined effects of obstacle avoidance and end-effector orientation maintenance constraints, the manipulator remains within certain joint angular velocity limits, ensuring stable operation. When joint angles method the limit range, adjustments are quickly made to reduce angular velocities, achieving smooth and stable operation, thus proving the effectiveness of the physical constraints.

Simulations demonstrate that the LMFC scheme effectively integrates physical constraints, obstacle avoidance, end-effector orientation maintenance, and a joint-drift-free strategy, optimizing performance in redundant manipulators, the Franka Panda. By leveraging additional degrees of freedom, it enhances flexibility, task performance, obstacle navigation, and force distribution. Adjusting constraint proportions as needed further boosts efficiency, coordination, and adaptability across various environments.

### 5.3. Physical Experiments

**Figure 18:**
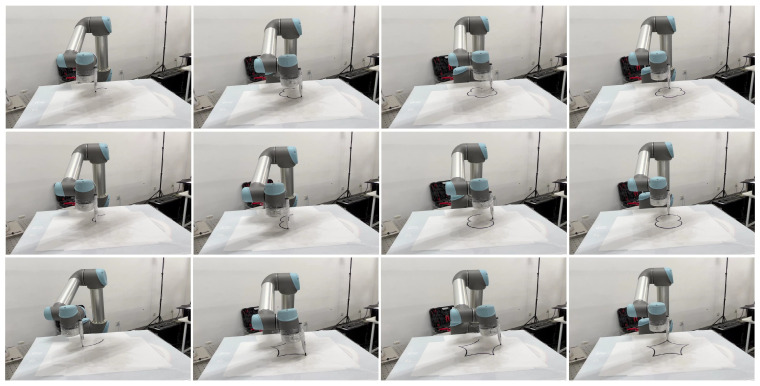
Physical demonstration of the trajectory tracking control for the robotic manipulator (end-effector drawing).

In physical experiments, the manipulator tracks a desired trajectory within 20 s using joint velocity signals. Real-time measurements from an integrated position sensor are used to adjust joint angles, velocities, and accelerations, ensuring precise and efficient trajectory tracking. The LMFC scheme effectively controls the manipulator in real-time for various trajectory tracking tasks. Figure 18 showcases practical demonstrations of the manipulator’s precise trajectory tracking in end-effector drawing and medical ultrasound scanning tasks.

**Figure 19:**
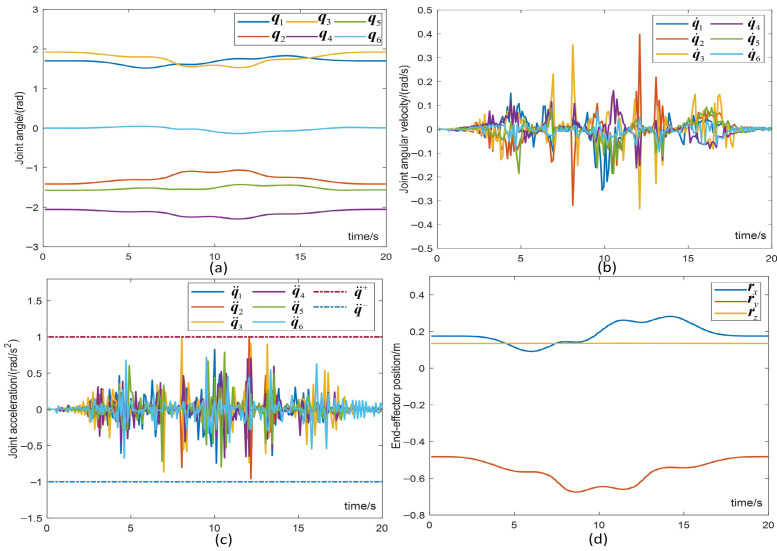
The joint motion and end-effector position of the robotic manipulator (pentagram trajectory tracking).

Figure 19 provides joint angles, velocities and accelerations, and end-effector positions during end-effector drawing tasks (as an example of a pentagram trajectory). Specifically, Figure 19a,b depict smooth joint angle and velocity variations, with the end-effector following a closed and smooth trajectory, ensuring successful task execution without joint angle drift. The return of final joints to their initial state confirms the achievement of the RMP and JDF performance optimization criteria. Position and velocity in joint space remain continuous and stable, with no changes in end-effector orientation. Figure 19c show well-constrained joint acceleration, nearing the boundary but remaining within acceptable limits. Figure 19d display the actual end-effector position, illustrating smooth and stable task execution. Particularly, *Z*-axis displacement remains constant, indicating stable contact force. This demonstrates effectiveness and practicality of the LMFC scheme in hybrid motion–force control for practical applications.

## 6. Conclusions

This paper presents a bioinspired LMFC framework that integrates adaptive learning with motion–force coordination to achieve precise, robust, and compliant manipulation in complex environments. By unifying kinematic control and adaptive learning within a TVQP formulation and equipping it with an RNN-based adaptive controller, the LMFC framework enables natural motion–force cooperation while estimating implicit kinematic parameters online and enforcing multi-level constraints, thereby ensuring stable and consistent performance under uncertainty. Compared with existing state-of-the-art controllers that rely on explicit dynamic modeling or dense sensing, the proposed framework demonstrates markedly superior robustness and adaptability. Simulations and physical experiments further validate the effectiveness and practicality of LMFC, achieving high-precision motion–force regulation with position errors on the order of $10^{-7}\;m$, contact-force steady-state errors around $10^{-4}\;N$, strict adherence to physical limits, reliable obstacle avoidance, near-zero orientation deviation, and minimal joint drift on the order of $10^{-3}\;\mathrm{rad}$. These results confirm that LMFC offers a biologically inspired foundation for adaptive and compliant robotic manipulation, significantly enhancing precision and safe interaction in complex environments. Future work will focus on extending the framework to manipulators with more complex kinematic structures, incorporating richer sensory modalities for enhanced perception and interaction, and advancing real-time adaptive control strategies suited for highly dynamic and uncertain environments, particularly under time delays or low actuation accuracy.

## Figures and Tables

**Table 1:** Comparisons among different controllers for robotic manipulators.

	Motion–Force Control Mode	Obstacle Avoidance	Physical Constraints	DLC	JDF	Effector Maintenance	Unknown Kinematic	Driven Signal	Physical Experiment
Ours	Motion–Force	Yes	Angle & Velocity & Acceleration	Yes	Yes	Yes	Yes	Velocity	Yes
[34]	Motion–Force	No	Angle & Velocity	No	No	No	No	Velocity	Yes
[13]	Motion–Force	No	Angle & Velocity	No	No	Yes	Yes	Velocity	Yes
[12]	Motion	Yes	Angle & Velocity	No	No	Yes	No	Velocity	Yes
[20]	Motion	No	Angle & Velocity & Acceleration	Yes	No	No	Yes	Velocity	Yes
[21]	Motion	No	Acceleration	Yes	Yes	No	No	Yes	Yes
[4]	Motion	No	Velocity	No	No	Yes	Yes	Velocity	Yes
[7]	Motion	No	Angle & Velocity	No	No	No	Yes	Velocity	Yes
[10]	Motion	Yes	Angle & Velocity	Yes	No	Yes	No	Angle	Yes
[14]	Motion	Yes	Angle & Velocity	No	No	No	No	Velocity	Yes
[15]	Motion–Force	No	Acceleration	Yes	No	No	No	Velocity	Yes
[16]	Motion	Yes	Velocity	No	No	Yes	Yes	Velocity	Yes
[17]	Motion	No	Velocity	No	No	No	Yes	Velocity	Yes
[18]	Motion	Yes	Velocity	No	No	No	No	Velocity	Yes
[35]	Motion	No	No	No	No	No	No	Velocity	Yes
[32]	Motion	No	Velocity	Yes	Yes	No	Yes	Angle	Yes
[33]	Motion	No	No	No	Yes	No	No	Velocity	Yes

**DLC (Decision-Level Constraint):** Indicates that both the control decision variable and its derivative are explicitly constrained, ensuring physically feasible, smooth, and stable control actions. Effector maintenance refers to maintaining the end-effector orientation. **JDF (Joint-Drift-Free) Criterion:** Ensures that terminal joint velocities and accelerations converge to zero, preventing drift accumulation and decoupling residual joint motion from task-space behavior. **Driven Signal:** Specifies the type of actuation command (e.g., velocity, torque, angle), clarifying the controller’s implementation pathway.

**Table 2:** Key Criteria in the LMFC–TVQP Formulation (Regarding Remark 4).

Criterion	Functional Role	Contribution to Multi-Constraint Coordination and System Performance
Physical Constraints	Ensures joint torques, velocities, and accelerations remain within safe and feasible limits	Maintains safety and feasibility, prevents hardware damage, and guarantees stable execution under physical limits
Obstacle Avoidance	Prevents collisions by dynamically adjusting trajectories	Enhances adaptability in cluttered environments and ensures task completion without collisions
End-Effector Orientation Maintenance	Maintains the desired orientation of the end-effector during manipulation	Preserves task accuracy and precision, especially in orientation-sensitive operations
Joint Angle Drift Elimination	Compensates for kinematic uncertainties and sensor drift	Improves long-horizon trajectory accuracy, ensures repeatability, and stabilizes manipulator performance

**Table 3:** The corresponding kinematic parameters for the assumed values of the actual Jacobian matrix.

Joint Number	α (°)	*a* (mm)	*q* (°)	*d* (mm)
1	90	0	$q_{1}$	89.2
2	0	−425	$q_{2}$	0
3	0	−392	$q_{3}$	0
4	90	0	$q_{4}$	109.3
5	−90	0	$q_{5}$	94.75
6	0	0	$q_{6}$	82.5

**Table 4:** Comparison of the joint angular drifts when considering (λ=6) and not considering (λ=0) the JDF criterion.

Joint Angular Drifts (rad)	λ=0	λ=6
$q_{1}\left(5\right)-q_{1}\left(0\right)$	−1.261	$-4.065\times 10^{-7}$
$q_{2}\left(5\right)-q_{2}\left(0\right)$	0.442	$7.732\times 10^{-7}$
$q_{3}\left(5\right)-q_{3}\left(0\right)$	−0.609	$1.053\times 10^{-6}$
$q_{4}\left(5\right)-q_{4}\left(0\right)$	0.206	$-2.899\times 10^{-6}$
$q_{5}\left(5\right)-q_{5}\left(0\right)$	0.244	$-1.197\times 10^{-6}$
$q_{6}\left(5\right)-q_{6}\left(0\right)$	−0.060	$3.427\times 10^{-7}$
${∥q\left(5\right)-q\left(0\right)∥}_{2}$	1.504	$3.439\times 10^{-6}$

**Table 5:** The corresponding kinematic parameters of the Franka Panda.

Joint Number	α (°)	*a* (mm)	*q* (°)	*d* (mm)
1	0	0	$q_{1}$	333
2	−90	0	$q_{2}$	0
3	90	0	$q_{3}$	316
4	90	82.5	$q_{4}$	0
5	−90	−82.5	$q_{5}$	384
6	90	0	$q_{6}$	0
7	90	88	$q_{6}$	0

**Table 6:** Minimum Parameter Values under Different Constraint Configurations.

Condition	$s_{\min}\;\left(m\right)$	$s_{o}\;\left(m\right)$	$s_{a}\;\left(m\right)$
Joint-drift-free scheme	0.037	0.05	0.1
Obstacle avoidance	0.093	0.05	0.1
End-effector orientation maintenance	0	0.05	0.1
Combined constraint	0.080	0.05	0.1

## Data Availability

Data are contained within the article.
